# Neural representations for multi-context visuomotor adaptation and the impact of common representation on multi-task performance: a multivariate decoding approach

**DOI:** 10.3389/fnhum.2023.1221944

**Published:** 2023-09-26

**Authors:** Youngjo Song, Wooree Shin, Pyeongsoo Kim, Jaeseung Jeong

**Affiliations:** ^1^Department of Bio and Brain Engineering, College of Engineering, Korea Advanced Institute of Science and Technology (KAIST), Daejeon, South Korea; ^2^Program of Brain and Cognitive Engineering, College of Engineering, Korea Advanced Institute of Science and Technology (KAIST), Daejeon, South Korea; ^3^Department of Brain and Cognitive Sciences, College of Life Science and Bioengineering, Korea Advanced Institute of Science and Technology (KAIST), Daejeon, South Korea

**Keywords:** context representation, sensorimotor adaptation, multi-task, structural learning, shared representation, meta-learning, multi-voxel pattern analysis (MVPA)

## Abstract

The human brain's remarkable motor adaptability stems from the formation of context representations and the use of a common context representation (e.g., an invariant task structure across task contexts) derived from structural learning. However, direct evaluation of context representations and structural learning in sensorimotor tasks remains limited. This study aimed to rigorously distinguish neural representations of visual, movement, and context levels crucial for multi-context visuomotor adaptation and investigate the association between representation commonality across task contexts and adaptation performance using multivariate decoding analysis with fMRI data. Here, we focused on three distinct task contexts, two of which share a rotation structure (i.e., visuomotor rotation contexts with −90° and +90° rotations, in which the mouse cursor's movement was rotated 90 degrees counterclockwise and clockwise relative to the hand-movement direction, respectively) and the remaining one does not (i.e., mirror-reversal context where the horizontal movement of the computer mouse was inverted). This study found that visual representations (i.e., visual direction) were decoded in the occipital area, while movement representations (i.e., hand-movement direction) were decoded across various visuomotor-related regions. These findings are consistent with prior research and the widely recognized roles of those areas. Task-context representations (i.e., either −90° rotation, +90° rotation, or mirror-reversal) were also distinguishable in various brain regions. Notably, these regions largely overlapped with those encoding visual and movement representations. This overlap suggests a potential intricate dependency of encoding visual and movement directions on the context information. Moreover, we discovered that higher task performance is associated with task-context representation commonality, as evidenced by negative correlations between task performance and task-context-decoding accuracy in various brain regions, potentially supporting structural learning. Importantly, despite limited similarities between tasks (e.g., rotation and mirror-reversal contexts), such association was still observed, suggesting an efficient mechanism in the brain that extracts commonalities from different task contexts (such as visuomotor rotations or mirror-reversal) at multiple structural levels, from high-level abstractions to lower-level details. In summary, while illuminating the intricate interplay between visuomotor processing and context information, our study highlights the efficiency of learning mechanisms, thereby paving the way for future exploration of the brain's versatile motor ability.

## Introduction

The human brain's remarkable versatility in motor skills enables individuals to adapt flexibly to various real-world situations, such as navigating streets, ascending stairs, and manipulating utensils (Wolpert et al., 2011; McDougle et al., 2016). This adaptive function stems from the formation of *context representations*, which help the brain encode and store information from different task contexts in a structured manner, improving the management of multiple visuomotor tasks (Badre et al., 2021; Heald et al., 2021). For instance, catastrophic interference—experiencing rapid and significant loss of prior knowledge when learning new information (McCloskey and Cohen, 1989)—in sensorimotor adaptation tasks can be mitigated (Kitago et al., 2013; Heald et al., 2021) by retaining latent memory traces with contextual information from prior adaptations and enabling more effective overcoming of interference and restoration or consolidation of previously learned skills when needed (Heald et al., 2021). Moreover, the brain utilizes *structural learning*, a process that extracts an invariant task-context representation that captures the commonalities across different tasks (e.g., a shared task structure), facilitating adaptation to new environments while reducing interference (see details in Braun et al., 2010a). In this sense, structural learning can be viewed as one form of meta-learning (i.e., learning to learn) since it involves extracting meta-information (e.g., task structure) that can be generalized across multiple tasks (due to the invariance of information), resulting in optimization of the learning process itself (Kemp and Tenenbaum, 2008; Kemp et al., 2010; Lansdell and Kording, 2019). Indeed, studies on visuomotor rotation contexts have shown that the motor control process can extract the common task structure inherent in rotational transformations, resulting in structure-specific facilitation of learning, interference reduction, and exploration (Braun et al., 2009; Genewein et al., 2015). In summary, the human brain's ability to form context representations and employ structural learning during this process contributes to its remarkable adaptability in various motor tasks.

Neuroimaging research has highlighted the importance of various brain regions in managing multiple representations related to different contexts: For instance, the prefrontal cortex (PFC) is involved in context representation and switching or updating them based on context transitions and sensory cues (Botvinick, 2008; Donoso et al., 2014; Marton et al., 2018; Anderson and Hulbert, 2021; Soltani and Koechlin, 2022); the hippocampus is responsible for encoding and retrieving context-dependent memories (Savin et al., 2014; Wikenheiser and Schoenbaum, 2016; Behrens et al., 2018); and the cerebellum also contributes to such memories (Batsikadze et al., 2022), while many other areas are implicated in certain aspects of context information. Although it is thought that the same brain regions can be largely shared for managing context information during sensorimotor tasks (Heald et al., 2023), more direct evaluation of sensorimotor tasks is relatively scarce. This is partly due to the complexity of distinguishing among sensory, movement, and task-context representations since the mapping between two fundamental elements (the sensory input and motor output) is dependent on the task context, leading to a complication in interpreting results on the context representations. However, a few significant multi-voxel pattern analysis (MVPA) studies address this issue, successfully assessing task-context representations during visuomotor adaptation: One study (Ogawa and Imamizu, 2013) demonstrated that the sensory and motor areas represent different visuomotor contexts; meanwhile, another study (Haar et al., 2015) indicates that direction-selective fMRI patterns in the posterior parietal cortex suggest the storage of the novel context in this region. However, these arguments deserve further confirmation due to their examination of limited visuomotor-adaptation scenarios and the need for more rigorous verification of their results; and importantly, the association between context representation commonality (possibly achieved by structural learning) and task performance is yet to be addressed.

MVPA can provide insights into the context representation commonality resulting from structural learning. If structural learning takes place, the brain should create a common representation across task contexts, reflecting the shared task structure (Braun et al., 2010a,b; Friedman, 2013). This common representation leads to a similar neural response to different task contexts, resulting in high representation similarity and low decoding accuracy in MVPA (Kriegeskorte et al., 2008; Hebart and Baker, 2018). Consequently, if structural learning enhances multi-task performance, a correlation between task performance and MVPA output (i.e., representation similarity or decoding accuracy) can be identified. Thus, utilizing MVPA may uncover crucial information about how structural learning contributes to multi-task performance by examining the relationship between representation commonality and performance outcomes.

In this study, we conducted a multi-context visuomotor-adaptation task and aimed to make a rigorous distinction of the neural representations of three different levels (i.e., visual, movement, and context) crucial for carrying out multi-context visuomotor adaptation, as well as the association between the commonality (i.e., shareability or invariance) of neural representations across task contexts and visuomotor-adaptation performance (i.e., task performance) using MVPA with fMRI data. We focused on three distinct visuomotor-adaptation contexts (i.e., task contexts), which are commonly examined in single-context visuomotor-adaptation studies. Two of the three were *visuomotor rotation contexts* (or “rotation contexts” for brevity): −90° and +90° rotations. In the −90° and +90° rotation contexts, the mouse cursor's movement was rotated 90 degrees counterclockwise and clockwise relative to the hand-movement direction, respectively. These two task contexts share the predominant task structure (i.e., rotation), allowing participants to benefit from learning a common structure when performing both contexts.

*A mirror-reversal context* (or “mirror context” for brevity) was included as an additional context among the three to further examine the relationship between contexts with limited shared structure. In the mirror-reversal context, the horizontal movement of the computer mouse was inverted, which is different from rotation. This creates a challenge in establishing a connection between learning the rotation structure and mirror-reversal context, leading to minimal benefit from structural learning (However, despite their differences, the mirror-reversal and rotation contexts share some minor structures, such as computer mouse sensitivity and the screen's viewing angle in front of the participant—since the same mouse and screen were used across task contexts—among other aspects. Therefore, the possibility that the mirror context benefits from a common representation—between mirror and rotation contexts—within the current experimental paradigm cannot be completely ruled out.). In sum, examining these three visuomotor-adaptation contexts allows for a more comprehensive understanding of multi-context representations and structural learning in the brain, considering both the representations shared between task contexts with significant structure (i.e., rotation) and those with only minor structure.

To rigorously differentiate various levels of neural representations (i.e., visual, movement, and task context) during the visuomotor process in the brain, we employed a conjunctional multivariate decoding analysis (Kahnt et al., 2010). In this analysis, by partitioning the data into training and test sets that enable generalization (i.e., transfer from a training set to a test set) using information exclusively from the desired representation level, it is possible to classify the desired level while minimizing the influence of information from other representation levels. Further details are provided in later sections. Based on the insight that similar neural responses between conditions restrict the classifier's decoding capability, we hypothesized that low context-decoding accuracy indicates a high context representation commonality due to structural learning, while the opposite scenario is indicated by high accuracy. Thus, a negative correlation between context-decoding accuracy and task performance can be expected if the common representation benefits performance. However, it is noteworthy that some theoretical works suggest that low context representation commonality can result in high performance, in contrast to the previous scenario (Musslick et al., 2017; Musslick and Cohen, 2019; Sagiv et al., 2020; Badre et al., 2021). This can occur when there is limited or no shared structure across task contexts (e.g., rotation vs. mirror), as relying on common resources during learning would lead to competition for processing and subsequent interference. Therefore, we remained open to this possibility, i.e., open to both positive and negative correlations.

The current study is mainly delineated into three *separate stages*. (1) Initially, a mass-univariate analysis was executed to explore the brain regions implicated in the visuomotor-adaptation task under investigation (i.e., which brain area is activated during the task). (2) Subsequently, a conjunctional multivariate decoding analysis was conducted to rigorously ascertain the specific brain area responsible for visual, movement, or task-context representations. (3) Finally, a correlation analysis between decoding accuracy and visuomotor-adaptation performance was conducted to examine the impact of context representation commonality on task performance.

## Materials and methods

### Subjects and ethics statement

A total of 23 participants (nine female participants, mean age: 22.9 years, standard deviation: 1.8 years), all right-handed, normal or corrected to normal vision, and with no reported history of neurological or psychiatric disorders, were recruited for this study. One female participant (age: 22) was excluded due to an inability to complete the entire MRI scanning session because of the numbness in her arm. Consequently, the final sample consisted of 22 subjects. All participants provided written informed consent, and the Institutional Review Board of the Korea Advanced Institute of Science and Technology approved the study (approval number: KH2019-164).

### Task description

Participants completed two consecutive experimental sessions over 2 days, with one session conducted each day. The initial session (day 1) was performed outside an MRI scanner, while the second session (day 2) took place inside the scanner. The initial session aimed to familiarize the subjects with the experiment and was carried out in a pseudo-MRI environment that simulated the conditions of the MRI scanner. In this simulated (i.e., pseudo-MRI) environment, participants were asked to lie on a bed and execute the given task while viewing the presented stimuli through a mirror positioned in front of them, which closely resembled the MRI setting. The *main analysis* in this study utilized data from the second session conducted inside the actual MRI scanner.

During the task (i.e., both on days 1 and 2), participants focused on a computer screen projected onto a projector screen via a mirror placed in front of them. On day 1 (i.e., the pseudo-MRI environment), the screen was positioned 1.35 m from their eyes, with dimensions of 0.4 × 0.3 m and a resolution of 800 × 600. On day 2 (i.e., inside the MRI scanner), the screen was positioned 2.7 m from their eyes, with dimensions of 0.8 × 0.6 m and a resolution of 800 × 600. Consequently, the viewing angle of the experimental stimuli remained consistent across both sessions.

**Figure 1:**
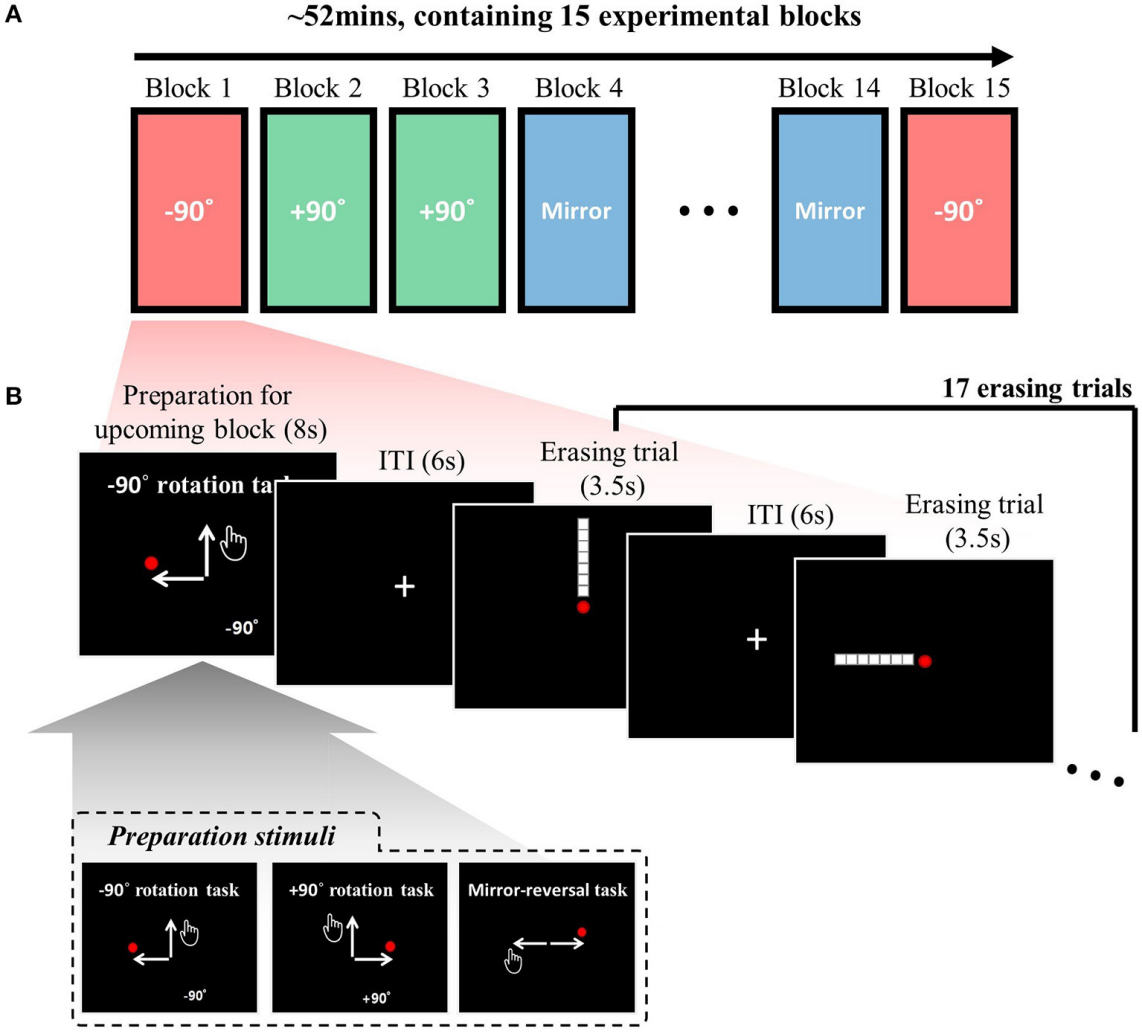
Task descriptions: **(A)** The experimental session, conducted each day, comprised 15 experimental blocks, with the adaptation context changing in each block. The three adaptation contexts (rotation −90°, rotation +90°, and mirror-reversal) were repeated five times each, resulting in a total of 15 experimental blocks. The order of the experimental blocks was permuted for every session. **(B)** A single experimental block consisted of 17 erasing trials [maximum duration of 3.5 s per trial and an inter-trial interval (ITI) of 6 s], including four distinct types: leftward, rightward, upward, and downward. Participants were instructed to sequentially erase a line composed of seven white square dots using a red circle cursor, following a specific direction (e.g., starting from the bottom in an upward erasing trial). The trial sequence was permuted using a type 1 index 1 continuous-carryover sequence, ensuring equal precedence and succession of each erasing direction, with distinct continuous-carryover sequences for each run and participant. At the beginning of each block, a preparation stimulus was presented, informing participants about the upcoming task context (i.e., −90°, +90°, or mirror). The inset displays the three distinct preparation stimuli corresponding to these three task contexts. For further details, refer to the Methods section.

Each session comprised 15 experimental blocks, consisting of three types of visuomotor-adaptation contexts (i.e., rotation −90°, rotation +90°, and mirror-reversal) repeated five times each (Figure 1A). The order of the experimental blocks was randomized for every session. The task involved three visuomotor-adaptation contexts: two visuomotor rotations (−90° and +90°) and a mirror-reversal. In the −90° rotation context, the mouse cursor's movement was rotated 90 degrees counterclockwise relative to the hand-movement direction (i.e., the physical computer mouse's movement; “movement direction” for brevity). Conversely, in the +90° rotation context, the mouse cursor's movement was rotated 90 degrees clockwise relative to the movement direction. In the mirror-reversal context, the horizontal movement of the computer mouse was inverted, while the vertical movement remained unchanged. These three contexts can be represented using the following 2x2 transformation matrices—transformations from the hand's position to the mouse cursor's coordination on the computer screen if we use left-handed cartesian coordinates:


$$\begin{array}{lll} T_{rot-90}\; & = & \;\left[\begin{array}{c} \cos\left(-\pi /2\right)\;-sin\left(-\pi /2\right)\\ \sin\left(-\pi /2\right)\;\cos\left(-\pi /2\right) \end{array}\right],\\ T_{rot+90}\; & = & \;\left[\begin{array}{c} \cos\left(\pi /2\right)\;-sin\left(\pi /2\right)\\ \sin\left(\pi /2\right)\;\cos\left(\pi /2\right) \end{array}\right],\\ T_{mirror}\; & = & \;\left[\begin{array}{c} -1\;0\\ \;0\;1 \end{array}\right]. \end{array}$$

The task context for the upcoming block is introduced through a preparation stimulus for 8 s (see Figure 1B). Thus, during this preparation period (8 s), the participants were allowed to understand and prepare for the upcoming visuomotor-adaptation context. This preparation stimulus includes a specific phrase that announces the type of visuomotor-adaptation context they will encounter next (either “−90° rotation task”, “+90° rotation task”, or “mirror-reversal task”) and a schematic diagram that visually illustrates the relationship between the movement of the participant's hand and the corresponding movement of the mouse cursor on the computer screen. The inset of Figure 1B displays the three different preparation stimuli used to cue each of the visuomotor-adaptation contexts. Following the completion of each experimental block (i.e., at the end of each block), the mean performance measures for the respective block are displayed in the form of the average number of erased dots and the time taken to erase them with a duration of 4 s.

Each experimental block comprised 17 erasing trials (Figure 1B), with four distinct types: leftward, rightward, upward, and downward erasing trials. Participants were required to erase a given line in a specified direction in each trial. For example, in an upward erasing trial (i.e., the first trial in Figure 1B), the line would be displayed in an upward direction relative to the mouse cursor (i.e., the red circle) at the center of the screen. The participant would then move the mouse cursor upward to erase the line. The lines were composed of seven white square dots (16 pixels wide and 16 pixels high) against a black background. The same arrangement of seven dots was utilized for all subjects. The mouse cursor was depicted as a red circle with a diameter of 12 pixels. The dot would be erased when the cursor's center overlapped with a target square dot. The line had to be erased sequentially, meaning participants could not erase dots from the middle. In an upward erasing trial, for instance, the line could only be erased starting from the bottom. In summary, in this study, “erasing” refers to the action of moving the mouse cursor over specific points in a sequential manner, causing them to disappear when hovered over.

**Figure 2:**
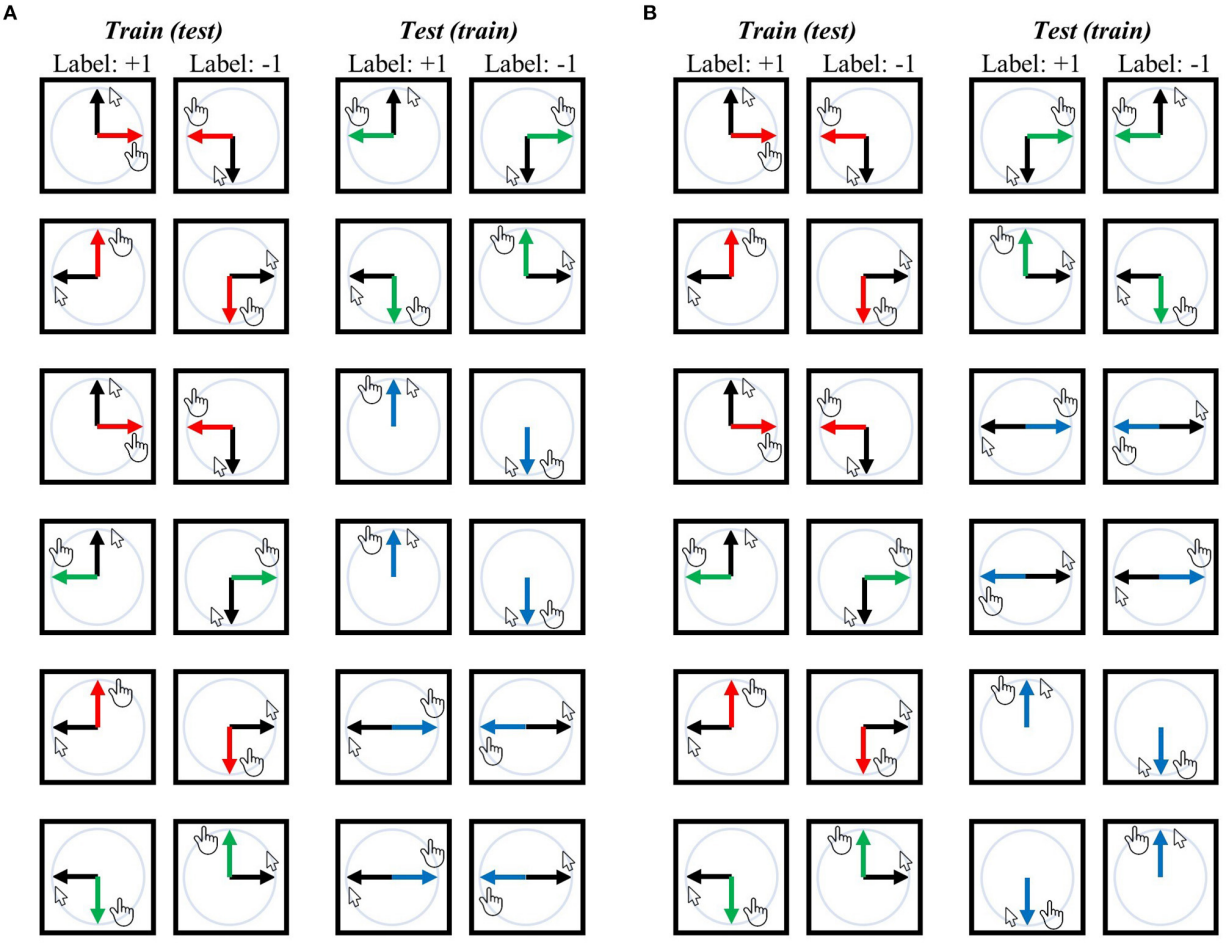
Training-test set pairs for decoding visual direction (i.e., visual representation) and movement direction (i.e., movement representation). The black arrows represent the designated mouse cursor's movement corresponding to the given line direction on the screen. The red arrows represent movement direction relative to the mouse cursor in the −90° rotation context, green arrows for the +90° rotation context, and blue arrows for the mirror context. **(A)** An exhaustive list of pairs for visual direction decoding independent of movement direction or task context, ensuring that the classifier (SVM) cannot rely on movement direction or task context when generalized from the training set to the test set because training and test sets possess different movement directions for corresponding labels and originate from distinct contexts. **(B)** An exhaustive list of training-test set pairs for decoding movement direction, where the classifier cannot depend on the visual direction or task context when generalization. In both analyses, any training set can potentially serve as a test set and vice versa, which results in 12 pairs for each decoding analysis. Accuracies from all 12 pairs were averaged, and then the chance (i.e., 50%) was subtracted from this averaged value, generating a single accuracy-minus-chance map of the entire brain for each participant. Note that the mirror context includes vertical motions (i.e., upward erasing and downward erasing); in these cases, the direction of the mouse cursor during erasing (i.e., visual direction) matches the actual direction in which the hand is moving (i.e., movement direction) [see the third and fourth pairs in **(A)** and the fifth and last pairs in **(B)**]. For further details, refer to the Methods section.

Note that in the mirror context, vertical motions such as upward and downward erasing are involved. During these motions, the direction of the mouse cursor on the screen (visual direction) aligns with the actual physical movement of the hand (movement direction). Specific examples of this alignment can be seen in the third and fourth pairs in Figure 2A and the fifth and last pairs in Figure 2B.

The sequence of trials was permuted for each experimental block. The order within each experimental block was structured as a type 1 index 1 continuous-carryover sequence (Aguirre, 2007). Consequently, the trials comprised four trials for each erasing direction (i.e., left, right, up, and down) along with one additional erasing trial (one of the four erasing directions), ensuring that each erasing direction preceded and succeeded every other erasing direction an equal number of times. It is noteworthy that the continuous-carryover sequence has been mainly utilized for studying repetition-suppression effects (i.e., the reduction in neural response that occurs when a stimulus or cognitive component is repeated) with fMRI (To avoid any confusion, it is also important to clarify that while these effects have sometimes been termed “adaptation,” in this study, we are using “adaptation” exclusively to refer to “behavioral adjustment for adapting to the perturbed environment in motor contexts.”). In the current study, the sequence was used to minimize the carryover effects (i.e., the influence of a prior stimulus on the response to the current or subsequent stimulus) in the decoding analyses. Since the continuous-carryover sequence ensures that each erasing direction preceded and succeeded every other erasing direction an equal number of times, it is possible to extract BOLD activation profiles of particular erasing direction while encompassing all possible carryover effects in every direction that could precede. By encompassing all carryover effects, the decoding analyses are more likely to utilize the BOLD activation profiles unique to that direction rather than being influenced by the repeated presentation of that direction or the carryover effects of previous directions, thereby eliminating potential biases from the analyses. A distinct continuous-carryover sequence was employed for each run, and a separate set of sequences was generated for every participant. The order template was as follows: (1, 2, 2, 3, 4, 4, 1, 1, 3, 3, 1, 4, 2, 4, 3, 2, 1). Each of the four erasing directions was randomly assigned to one of the four numbers to produce the trial sequence.

Prior to the experiment, participants were informed that they would engage in three visuomotor-adaptation contexts (i.e., rotation −90°, rotation +90°, and mirror-reversal) in a given environment (i.e., day 1: in a pseudo-MRI environment; and day 2: inside an MRI scanner) using a computer mouse. They were provided with an explanation of how the movement mapping between the mouse cursor and their hand would vary (i.e., an explanation about the three distinct contexts: rotation −90°, rotation +90°, and mirror-reversal). Participants were instructed that their objective was to erase a given stimulus as quickly as possible, with a maximum erasing time of 3.5 s. Thus, if the maximum erasing time (i.e., 3.5 s) was surpassed, the trial would be terminated irrespective of the participants' completion status in erasing the stimulus. On the other hand, if the participants completed the trial before the maximum erasing time was reached, the display would change to the fixation screen, and it would remain on this screen until the maximum erasing time had passed. To discourage reckless movements (such as aggressive wiping), they were cautioned that excessive force might cause the computer not to recognize the movement of the mouse.

Following the instructions, participants comfortably lay down in the MRI scanner, and their heads were positioned using a head retention device to minimize potential head movement. A mouse pad was placed under the subjects' right pelvis, and they held an MRI-compatible mouse (Nata Technologies) with their right hand. We allowed the participants to freely move the mouse with no applied transformations (i.e., unaltered movement: no rotation and no mirror-reversal) to familiarize themselves with using the mouse while lying down. Subsequently, we displayed five numbers on the screen (one through four positioned at the four corners and five at the center) and assessed whether they could reach any number on the screen using the normal mouse with no applied transformations. Once they confirmed that using the mouse was comfortable, the experiment commenced.

### Task performance

In this analysis, task performance for each trial was defined as the “*erasing speed*,” which represented the number of dots erased per second by the participant and served as an indicator of their proficiency in performing the given visuomotor-adaptation tasks. This quantity is calculated by the following formula:


$$\begin{array}{l} \left(\text{erasing speed}\right)\;=\;\frac{\left(\text{number of erased dots}\right)}{\left(\text{trial duration}\right)}. \end{array}$$

### MR imaging and pre-processing

For each participant, a single MRI run was conducted for the entire experimental session of 2nd day using a 3.0T Siemens Verio scanner. Functional images were acquired through a GE-EPI sequence, producing 3D functional images with a 3 × 3 × 3mm resolution for planar images (TR = 2300 ms, TE = 30ms, flip angle = 90 degrees, FOV = 166 × 163 × 126 mm). Structural images were obtained before the functional runs, using a 3D-MPRAGE sequence to acquire a structural image with a 1 × 1 × 1mm resolution for the entire brain.

Functional images were pre-processed using SPM12. For mass-univariate analysis, spatially aligned, unwarped (without field-map), slice-timing corrected, spatially normalized (using a deformation field obtained from the unified segmentation of the structural image), and spatially smoothed (using an 8-mm FWHM 3-D Gaussian kernel) functional images were generated. Meanwhile, for multivariate pattern analysis, spatially aligned, unwarped (without field-map), slice-timing corrected, and spatially smoothed (using a 2-mm FWHM 3-D Gaussian kernel) functional images were produced. Outlier scans (framewise displacement more than 0.9 mm or global BOLD signal changes more than 5 standard deviations) were detected from the realigned and slice-timing corrected functional images using the ART toolbox implemented in CONN toolbox (Whitfield-Gabrieli and Nieto-Castanon, 2012; Nieto-Castanon, 2020).

### Mass-univariate analysis

SPM12 was used for a mass-univariate analysis. In the first-level analysis, the general linear model (GLM) incorporated several types of task-related regressors: (1) erasing-related, (2) preparation stimulus for presenting upcoming task context, (3) score presentation, and (4) instruction stimulus for presenting the termination of each experimental block. For erasing-related regressors, three box-car regressors were constructed for erasing trials within each of the three visuomotor-adaptation contexts (i.e., rotation −90°, rotation +90°, and mirror-reversal) and convoluted with the hemodynamic response function. In some trials, participants explored the mouse cursor too much during erasing, possibly due to confusion about the currently required task information (e.g., task rule or adequate visuomotor mapping). To reduce the effect of such outlier trials, we omit the trials where the participants move their mouse cursors further than the one-and-a-half line length (i.e., 1.5 × 7 (dots) × 16 (pixels) = 168 pixels) when constructing the erasing-related regressors. Then, we add outlier-trial regressors (i.e., one regressor for each outlier trial) in the GLM as confounding regressors.

Confounding regressors further included movement-related regressors (i.e., six motion regressors and their gradients) and outlier spike regressors (i.e., one regressor for each outlier scan), where the outlier scans were flagged in the pre-processing pipeline (see previous section). The BOLD time series were high-pass filtered (1/128 Hz cutoff) to eliminate low-frequency noise and signal drift.

Group analysis was conducted using the beta values of the relevant regressors: i.e., (1) the erasing-related beta values for task activation analysis for each context; and (2) the preparation beta value for analysis of activation during preparing upcoming visuomotor adaptation. That is, this analysis focuses on identifying the areas where activation increased—compared to the implicit baseline of the fMRI signal—during (1) erasing—i.e., motor execution—or (2) preparing upcoming visuomotor-adaptation context. The random-field theory was used to control the family-wise error.

### Multivariate decoding analysis 1: decoding visual direction independent of movement direction or task context

In the first-level analysis, a general linear model was constructed per-experimental-block basis, i.e., incorporating task-related regressors for each block. For each block, four erasing-related regressors were included for each erasing direction (i.e., left, right, up, and down) and an additional null regressor for the first erasing trial. Outlier trials were excluded when constructing erasing-related regressors and added as separate outlier-trial regressors (as described in the previous section). Consequently, each block contained four erasing-related regressors (i.e., left, right, up, and down), one null regressor, one preparation regressor, one score-presenting regressor, and one session-termination regressor. Confounding regressors were the same as those in the previous mass-univariate analysis (i.e., accounting for movement and outlier scans). A multi-voxel pattern decoding analysis was then carried out using *the Decoding Toolbox* (Hebart et al., 2015). The support vector machine, implemented in the LIBSVM package, was used as a decoding algorithm (i.e., classifier). Beta maps from the first-level GLM, corresponding to the erasing-related regressors (i.e., four regressors per block), served as features for classification. Searchlight analysis was employed, encompassing the entire brain within searchlight spheres (9 mm radius).

To examine the neural representation of visual direction (i.e., visual representation) independent of movement direction or task context, a classifier was trained to differentiate between upward and downward (or between leftward and rightward) visual directions of a single context (e.g., −90° rotation) and subsequently tested on its ability to classify the upward and downward (or leftward and rightward) visual directions of another task (e.g., +90° rotation or mirror) (refer to Figure 2A for a comprehensive overview of training-test set pairs). In this scenario, the classifier cannot rely on movement direction information as the corresponding movement directions in the training and test sets are different. For instance, in the training-test set pairs illustrated at the top of Figure 2A, the training set demonstrates that the corresponding movement for the upward visual direction is rightward, while for the downward visual direction, it is leftward. In contrast, in the test set, the corresponding movement for the upward visual direction is leftward, and for the downward visual direction, it is rightward. Moreover, the classifier cannot rely on visuomotor-adaptation context information as the training and test sets originate from different contexts.

Then, the accuracies from all the training-test set pairs were averaged, and then, the chance (i.e., 50%) was subtracted from this averaged value, yielding a single accuracy-minus-chance map of the entire brain for each participant. The accuracy-minus-chance maps for each participant were spatially normalized using a deformation field obtained from the unified segmentation of the structural image, spatially smoothed (using an 8mm FWHM 3-D Gaussian kernel), and then used in the group-level analysis (i.e., t-test). In the group-level analysis, threshold-free cluster enhancement (TFCE) was used to control the family-wise error (Smith and Nichols, 2009), where a distribution of maximum TFCE values was generated by 5000 permutations.

### Multivariate decoding analysis 2: decoding movement direction independent of visual direction or task context

A comparable analysis investigated the neural representation of hand-movement directions (i.e., movement representation) independent of visual direction or task context. To this end, a classifier was trained to differentiate between upward and downward (or between leftward and rightward) movement directions of a single context (e.g., −90° rotation) and subsequently tested on its ability to classify the upward and downward (or leftward and rightward) movement directions of another context (e.g., +90° rotation or mirror) (refer to Figure 2B for a comprehensive overview of training-test set pairs). Consequently, the classifier could not rely on visual direction or adaptation context information, as corresponding visual directions and visuomotor-adaptation context differ between the training and test sets (see previous section for related arguments). The accuracies from all training-test set pairs were averaged, and then the chance (i.e., 50%) was subtracted from this averaged value, yielding a single accuracy-minus-chance map per participant. Then, the accuracy-minus-chance maps were spatially normalized, spatially smoothed (using an 8-mm FWHM 3-D Gaussian kernel), and used in group-level analysis (i.e., t-test). The TFCE was used to control the family-wise error, where a distribution of maximum TFCE values was generated by 5000 permutations.

### Multivariate decoding analysis 3: decoding task context independent of visual direction or movement direction

A similar analysis was conducted to investigate the neural representation of task context (i.e., task-context representation) independent of visual or movement directions. To account for the considerable behavioral difference between rotation and mirror contexts (see Behavior results section), two separate analyses were conducted: one decoding the two rotation contexts (−90° vs. +90°) and the other decoding rotation and mirror contexts (i.e., −90° vs. mirror and +90° vs. mirror) while including the absolute value of performance difference between rotation and mirror contexts as a confounding variable in the latter.

**Figure 3:**
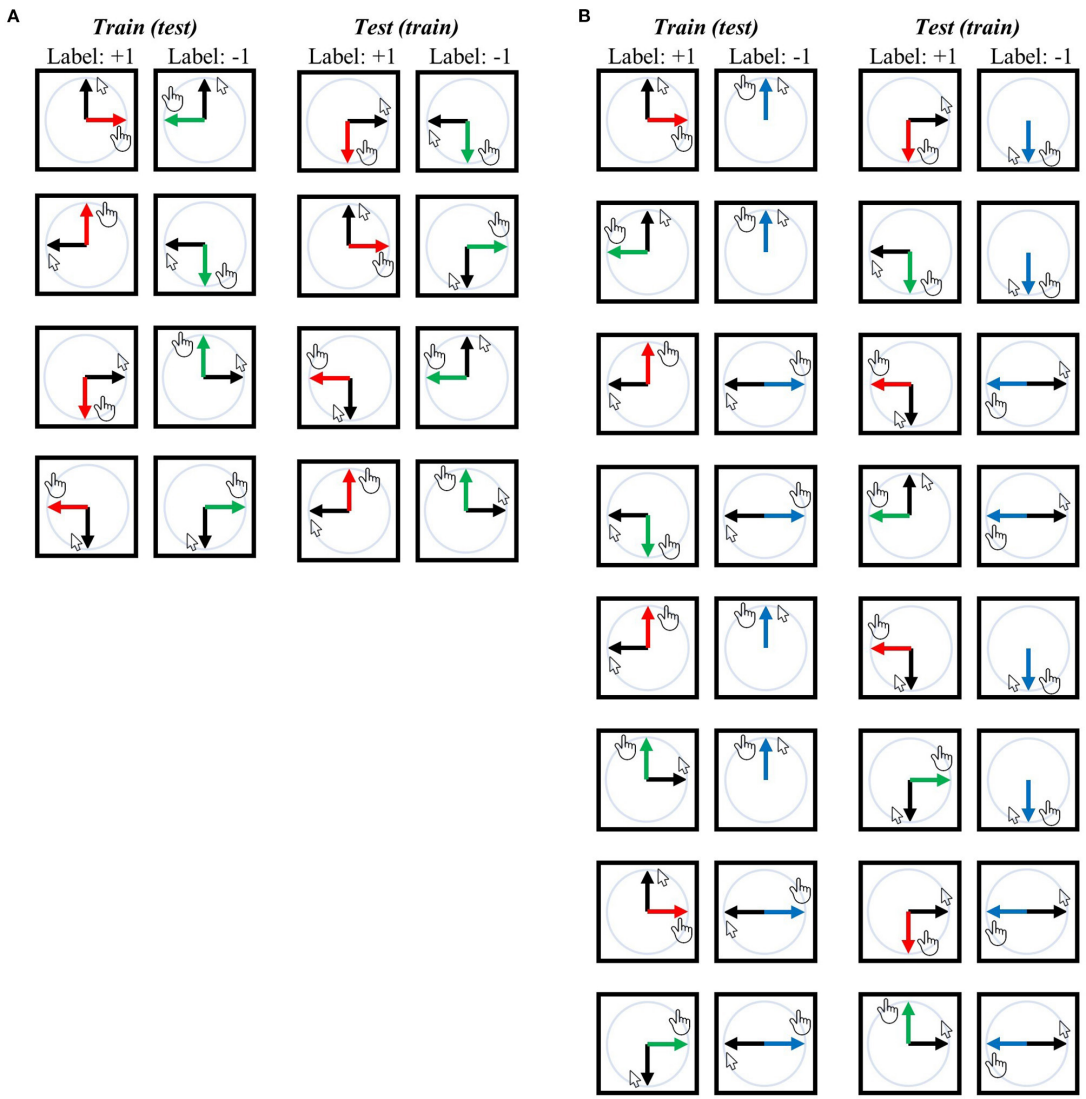
Training-test set pairs for decoding task contexts (i.e., task-context representation). The black arrows represent the designated mouse cursor's movement corresponding to the given line direction on the screen. The red arrows represent movement direction relative to the mouse cursor in the −90° rotation context, green arrows for the +90° rotation context, and blue arrows for the mirror context. **(A)** An exhaustive list of pairs for “−90° vs. +90°” task-context decoding independent of visual or movement direction. **(B)** An exhaustive list of pairs for “rotation vs. mirror” task-context decoding independent of visual or movement direction. Both analyses ensure that the classifier (SVM) cannot rely on the visual direction or movement direction when generalized from the training set to the test set because training and test sets possess different visual or movement directions for corresponding labels. Any training set can potentially serve as a test set and vice versa, resulting in a total of eight pairs for **(A)** and 16 pairs for **(B)**. For further details, refer to the Methods section.

In the first decoding analysis (i.e., −90° vs. +90°), a classifier was trained to differentiate these two task contexts using the same visual or movement direction, and then it was tested using different visual or movement directions. Thus, the classifier could not rely on the visual direction or movement direction when decoding task contexts (see previous sections for related arguments). For example, in the training-test set pairs illustrated at the top of Figure 3A, the classifier was trained using a set consisting of the upward visual direction of the −90° context and the upward visual direction of the +90° context; subsequently, it was tested to classify the −90° and +90° contexts with a data set consisting of the rightward visual direction of the −90° context and the leftward visual direction of the +90° context. A comprehensive overview of training-test set pairs can be found in Figure 3A.

Note that each training-test set pair was further subdivided using a leave-two-block-out cross-validation (CV) scheme, as temporally adjacent BOLD signals (i.e., within a single experimental block) may exhibit similarity irrespective of the actual neural signal present. For each CV fold, two temporally adjacent experimental blocks with different labels were excluded from the training set, resulting in each training-test pair being divided into five separate CV folds. Consider an example where we perform decoding analysis with the first pair shown in Figure 3A: In particular, when the training set consists of data from an upward visual direction trial with either −90° or +90° rotation, while the testing set includes downward movement direction data with rotations of −90° and +90°. As each task context was repeated five times (in other words, five experimental blocks), there are five data points for each label (−90° or +90°) in both the training and testing sets. We then further broke down this training-test pair into five smaller pairs. Let us number the blocks as −90° #1 to #5 and +90° #1 to #5, where the number signifies the sequential order of execution. In the first of these sub-pairs, the test set comprises the downward movement direction data of block −90° #1 and block +90° #1, while the training set includes the upward visual direction data of block −90° #2–#5 and block +90° #2–#5. In the second sub-pair, the test set consists of the downward movement direction data of block −90° #2 and block +90° #2, while the training set includes upward visual direction data of block −90° #1, #3, #4, #5 and block +90° #1, #3, #4, #5. The remaining sub-pairs were divided in the same manner. This process of subdivision was applied to all training-test set pairs illustrated in Figure 3A, resulting in five sub-pairs (i.e., five CV fold) for every training-test set pair depicted in the same figure.

The accuracies from all CV folds of the training-test set pairs were averaged, and then, the chance (i.e., 50%) was subtracted from this averaged value, yielding a single accuracy-minus-chance map for each participant. Then, the accuracy-minus-chance maps were spatially normalized, spatially smoothed (using an 8-mm FWHM 3-D Gaussian kernel), and used in the group-level analysis (i.e., t-test). The TFCE was used to control the family-wise error, where a distribution of maximum TFCE was generated by 5000 permutations.

The same analysis was performed in the second context-decoding analysis (i.e., rotation vs. mirror). An overview of training-test set pairs can be seen in Figure 3B. Those pairs were also subdivided using a leave-two-block-out CV scheme as we described above. The accuracies from all CV folds from all the training-test set pairs were averaged, and then the chance (i.e., 50%) was subtracted from this averaged value, yielding a single accuracy-minus-chance map for each participant. Then, the accuracy-minus-chance maps were spatially normalized, spatially smoothed (using an 8-mm FWHM 3-D Gaussian kernel), and used in the group-level analysis. Since a significant difference in task performance between rotation and mirror contexts exists, it is challenging to ascertain whether decoding significance arises from distinct context representations or differing kinematics (i.e., different movement speeds between rotation and mirror contexts) in this analysis. To address this, a group-level GLM analysis with an additional confound was conducted: The absolute value of the difference between the mean rotation context and mean mirror context performances was included as a confounding variable. Specifically, the group-level GLM analysis incorporated the confounding variable without centering, and the statistical test was executed for the intercept variable (i.e., constant regressor), thus corresponding to a t-test that considers the confound. The TFCE was used to control the family-wise error, where a distribution of maximum TFCE values was generated by 5000 permutations.

### Multivariate decoding analysis 4: correlation between context-decoding accuracy and task performance

For this correlation analysis, additional decoding analyses were conducted. The methodology was fundamentally similar to the prior context-decoding analyses, with two separate decoding analyses conducted: one for decoding −90° vs. +90° and the other for decoding rotation vs. mirror. The training-test set pairs remained consistent with the previous analyses. However, the leave-two-block-out CV was not employed to maximize the subject difference in accuracy as the focus was on the difference in accuracy rather than the exact value. Consequently, the training-test set pairs were not subdivided. These analyses resulted in two accuracy-minus-chance maps for each participant (i.e., one for −90° vs. +90° and the other for rotation vs. mirror). The accuracy-minus-chance maps were spatially normalized, spatially smoothed (using an 8mm FWHM 3-D Gaussian kernel), and used in the group-level analysis (i.e., GLM analysis).

First, a regression (i.e., correlation) analysis was performed to examine the relationship between the mean rotation context performance and the decoding accuracy for the “−90° vs. +90°” condition. Second, a correlation analysis was conducted to investigate the association between the mean mirror-context performance and the decoding accuracy for the “rotation vs. mirror” condition. In the second analysis, the absolute value of the difference between the mean rotation context and mean mirror context performances was included as a confounding variable. The TFCE was used to control the family-wise error, where a distribution of maximum TFCE values was generated by 5000 permutations.

### Multivariate decoding analysis 5: decoding task context from the preparation-period data

Two separate decoding analyses were conducted to decode task context from the preparation-period data (i.e., during the presentation of the upcoming visuomotor-adaptation context), following a procedure similar to the previous context-decoding analyses: one for decoding −90° vs. +90° and the other for decoding rotation vs. mirror. The beta maps of the preparation-related regressors in the 1st level general linear model (GLM) were utilized as features. In the first decoding analysis (i.e., −90° vs. +90°), a leave-two-out cross-validation (CV) approach was employed, excluding two temporally adjacent data points with different labels from the training set and resulting in five separate CV folds. The accuracies from all five CV folds were averaged, and then the chance (i.e., 50%) was subtracted from this averaged value, yielding a single accuracy-minus-chance map for each participant. In the second decoding analysis (i.e., rotation vs. mirror), leave-two-out CV was employed for both “−90° vs. mirror” and “+90° vs. mirror” decoding analyses, generating five separate CV folds for each. The accuracies from all 10 CV folds (five for each) were averaged, and then the chance (i.e., 50%) was subtracted from this averaged value, generating a single accuracy-minus-chance map for each participant.

The accuracy-minus-chance maps were spatially normalized, spatially smoothed (using an 8mm FWHM 3-D Gaussian kernel), and used in the group-level analysis (i.e., t-test). The TFCE was used to control the family-wise error, where a distribution of maximum TFCE was generated by 5000 permutations.

## Results

### Behavior results

**Figure 4:**
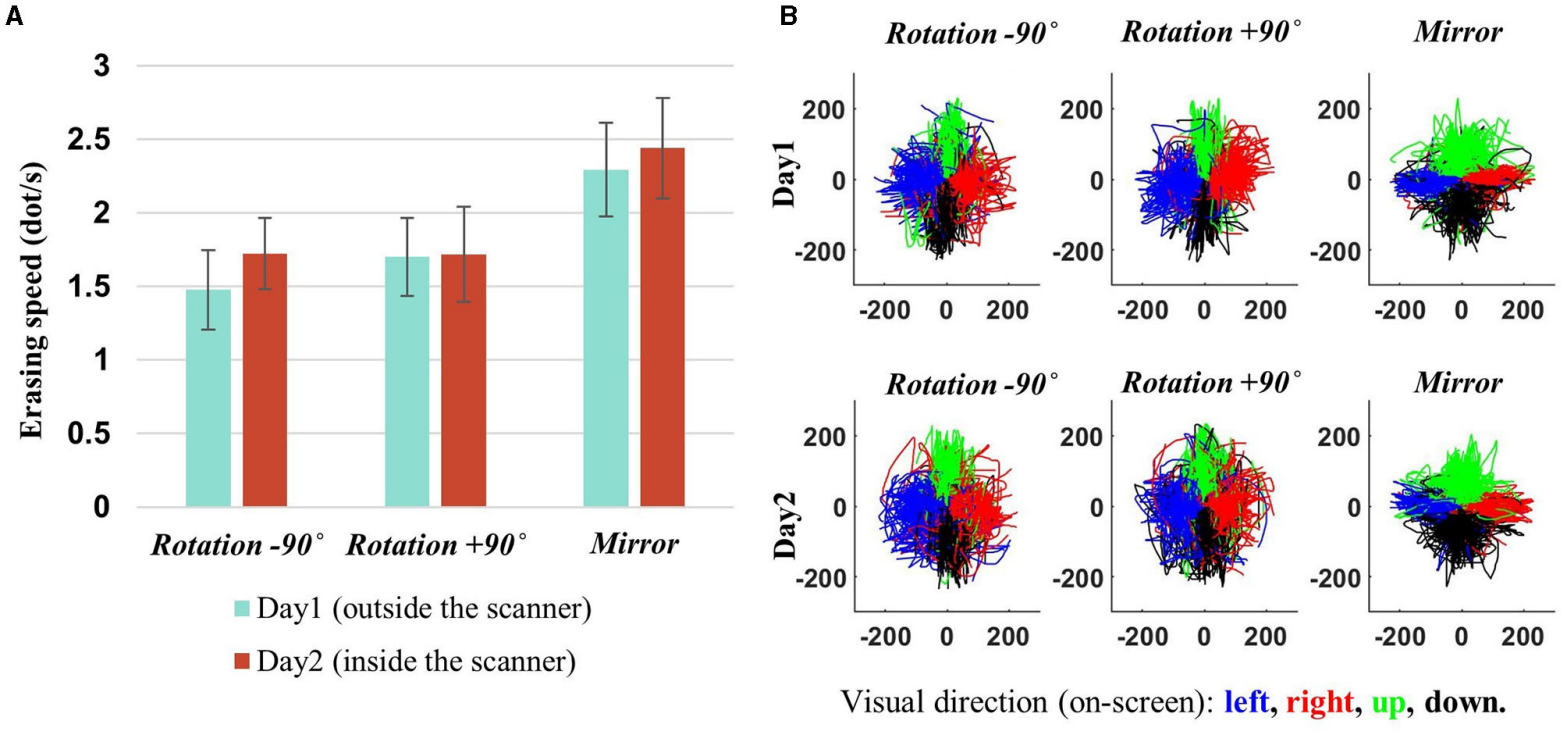
Behavioral results. **(A)** Subject-averaged erasing speed (i.e., performance) for each adaptation context, with error bars denoting 95% confidence intervals. A slight improvement can be observed on day 2. Performance measures surpassing zero demonstrate that participants successfully navigated the mouse cursor in the intended direction for each adaptation context. **(B)** The mouse cursor trajectories for all participants are depicted in the coordinates of the mouse cursor on the computer screen, with each color (blue, red, green, and black) representing a specific visual direction, indicating the intended direction for mouse cursor movement (left, right, up, and down). That is, these trajectories are derived from transformations of the hand's position into the mouse cursor's coordinates on the screen. As illustrated in the figure, the alignment of the trajectory directions with the given visual directions signifies that the participants effectively adapted to all the environmental changes (−90°, +90°, and mirror) by generating appropriate movements (e.g., generating upward movement when the leftward visual direction presents during −90° rotation context).

Participants exhibited a slight improvement in task performance (i.e., erasing speed) on the second day compared to the first day, with an average erasing speed of 1.7 dots per second for the rotation contexts (i.e., −90° and +90°) and 2.4 dots per second for the mirror-reversal context (Figure 4A). The task performance of the two rotation contexts became more comparable on the second day than the first day (−90° vs. +90°–day 1: p~0.1886, day 2: p~ 0.9870, Wilcoxon signed-rank test), while the task performance of the mirror-reversal context was significantly higher than the rotation contexts on both days (−90° vs. mirror—day 1: p~0.0002, day 2: p~0.0017; +90° vs. mirror—day 1: p~0.0010, day 2: p~0.0012, Wilcoxon signed-rank test). The task-performance measure exceeding zero for all three contexts signifies that the participants effectively moved the mouse cursor in the designated direction during each visuomotor-adaptation context. Indeed, for all task contexts, it was observed that they generally adhered to the requisite directional trajectory for erasing, indicating effective movement in the intended direction (Figure 4B).

### BOLD activation during erasing and preparation periods

**Figure 5:**
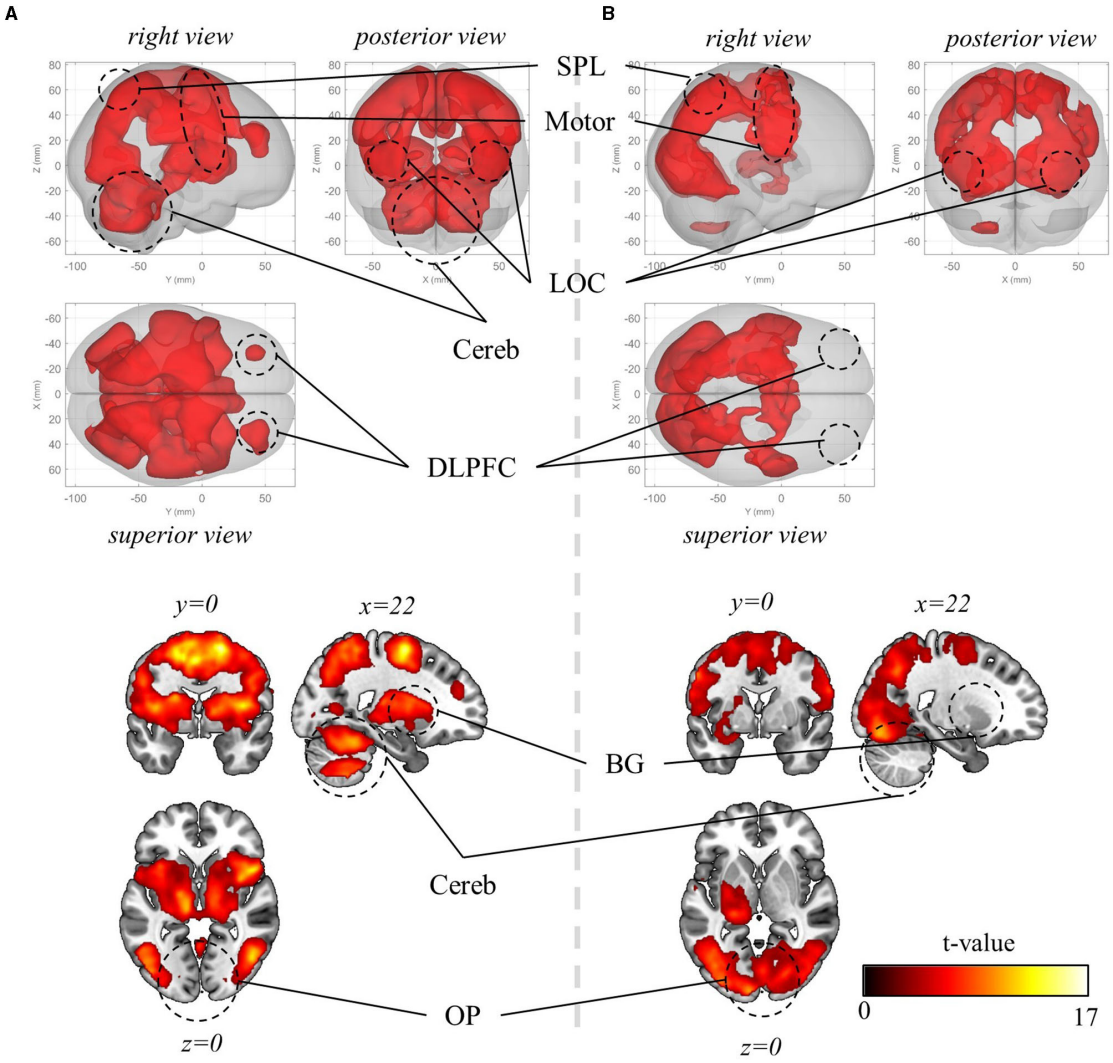
Mass-univariate analysis results. **(A)** Significant BOLD activation during visuomotor rotation +90° context (p_FWE_ < 0.05, corrected for the whole brain, cluster-level correction with uncorrected threshold *p* < 0.001, *n* = 22, one-sided, random-field theory). The BOLD activation during rotation −90° and mirror-reversal tasks is identical to the one during rotation +90° context, thus not depicted here. **(B)** Significant BOLD activation during the preparation period (p_FWE_ < 0.05, corrected for the whole brain, cluster-level correction with uncorrected threshold *p* < 0.001, *n* = 22, one-sided, random-field theory). In this period, the orientation stimulus, which informed participants of the context they would perform in the upcoming experimental block, was displayed at the beginning of the corresponding block. OP, occipital pole; LOC, lateral occipital cortex; SPL, superior parietal lobule; Motor, motor area that encompasses primary motor area, premotor area, supplementary motor area; Cereb, cerebellum; BG, basal ganglia; DLPFC, dorsolateral prefrontal cortex. For both **(A)** and **(B)**, the upper panels present a glass-brain view, while the lower panels present a sectional view. The color scales for both **(A)** and **(B)**.

Initially, mass-univariate analysis was conducted to examine the brain regions activated during visuomotor adaptation. Within all three visuomotor-adaptation contexts (−90°, +90°, and mirror), there was a significant increase in BOLD activation in various visuomotor areas during erasing (p_FWE_ < 0.05, corrected for the whole brain, cluster-level correction with uncorrected threshold *p* < 0.001, *n* = 22, one-sided, random-field theory). These areas encompass the lateral occipital cortex (LOC) (comprising superior and inferior parts), primary sensory area (S1), primary motor area (M1), premotor area (PM), supplementary motor area (SMA), posterior parietal area (PPA) (e.g., superior and inferior parietal lobules and precuneus), basal ganglia (BG), cerebellum, and dorsolateral prefrontal cortex (DLPFC) (Figure 5A). Notable differences were not observed between the activated regions across the visuomotor-adaptation contexts.

Then, we explored the activated regions during the preparation period—when the information about visuomotor-adaptation contexts (i.e., rotation +90°, rotation −90°, or mirror-reversal) was presented (i.e., before performing each session). This is when participants are first provided with information about the visuomotor-adaptation context of the upcoming session, allowing them to prepare or deduce the visuomotor mapping necessary for performing the session without the need for movement execution. Intriguingly, the activation region (p_FWE_ < 0.05, corrected for the whole brain, cluster-level correction with uncorrected threshold *p* < 0.001, *n* = 22, one-sided, random-field theory) largely coincided with the activated region during task performance, encompassing LOC, S1, M1, PM, SMA, PPA, anterior cingulate cortex (ACC), and occipital pole (OP) (Figure 5B). The major distinction was the substantially reduced or absence of significant activation in the DLPFC, BG, and cerebellum, while the OP exhibited more significant activation, indicating their distinctive roles between task preparation and real-time visuomotor control.

### Decoding results for visual, movement, and task-context representations

**Figure 6:**
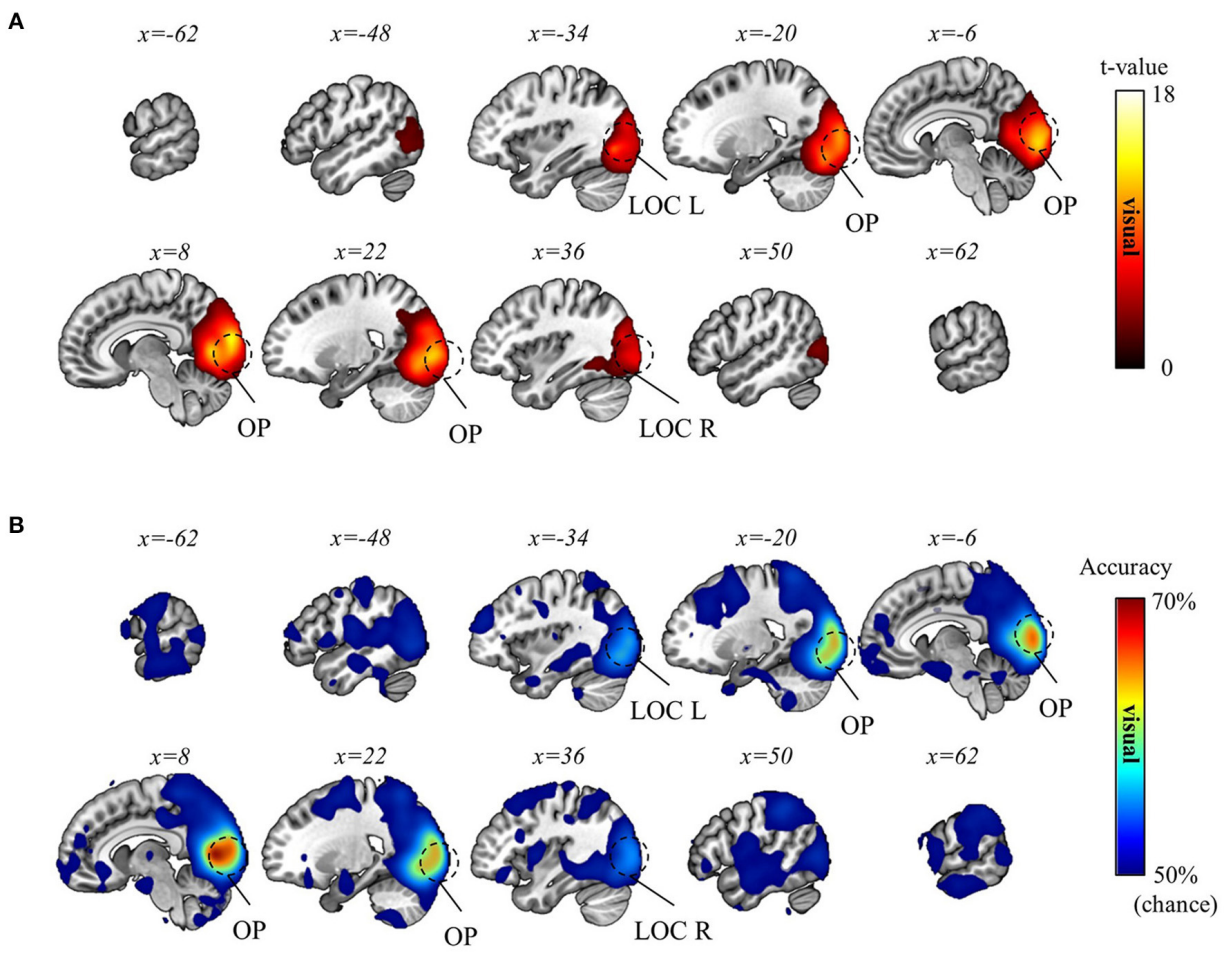
Visual direction decoding results. **(A)** A t-value map from the group-level analysis of searchlight decoding analysis for visual representations (i.e., visual direction) is presented. Only significant voxel t-values are displayed (p_FWE_ < 0.05, corrected for the whole brain, *n* = 22, one-sided, TFCE). **(B)** A subject-averaged accuracy map from the group-level analysis of searchlight decoding analysis for visual representations (i.e., visual direction) is presented. Only accuracies that are greater than chance are displayed (>50%). OP, occipital pole; LOC, lateral occipital cortex; L, left; R, right.

**Figure 7:**
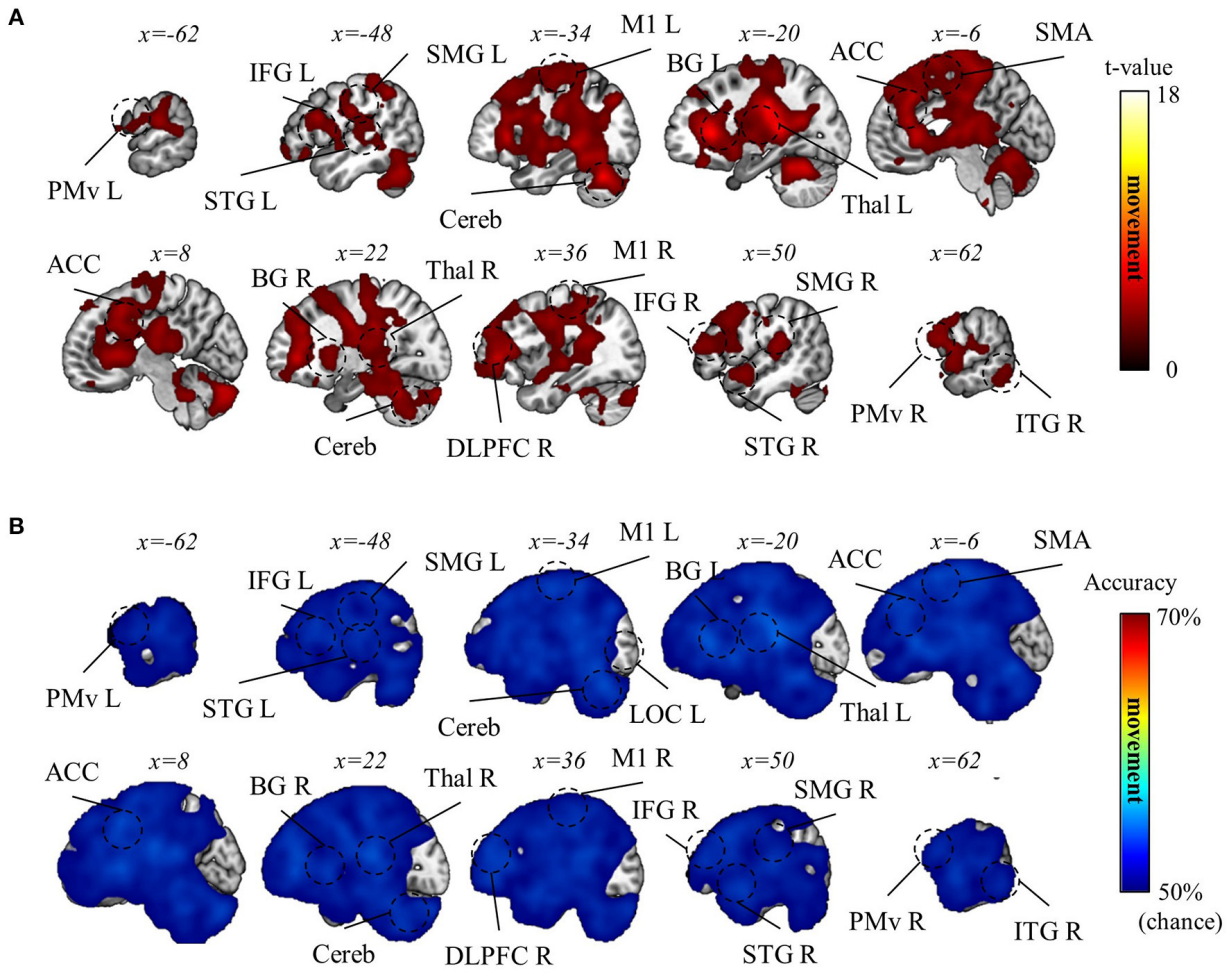
Movement direction decoding results. **(A)** A t-value map from the group-level analysis of searchlight decoding analysis for movement representations (i.e., movement direction) is presented. Only significant voxel t-values are displayed (p_FWE_ < 0.05, corrected for the whole brain, *n* = 22, one-sided, TFCE). **(B)** A subject-averaged accuracy map from the group-level analysis of searchlight decoding analysis for movement representations (i.e., movement direction) is presented. Only accuracies that are greater than chance are displayed (>50%). PreCu, precuneus; SPL, superior parietal lobule; SMG, supramarginal gyrus; M1, primary motor area; PMv, ventral premotor area; SMA, supplementary motor area; Cereb, cerebellum; BG, basal ganglia; Thal, thalamus; DLPFC, dorsolateral prefrontal cortex; IFG, inferior frontal gyrus; ACC, anterior cingulate cortex; OFC, orbitofrontal cortex; STG, superior temporal gyrus; ITG, inferior temporal gyrus; L, left; R, right.

Subsequently, we aimed to differentiate the (1) visual, (2) movement, and (3) task-context representations. As detailed in the Methods section, within the current experimental paradigm, (1) the visual representation denotes the line directions displayed on the screen, which also corresponds to the intended and actual cursor movement in the visual field; (2) the movement representation denotes the intended and actual direction of the computer-mouse (i.e., hand) movement on the mouse pad; and (3) the task-context representation denotes the three distinct visuomotor-adaptation contexts (i.e., −90°, +90°, and mirror). For this differentiation, we employed a conjunctional analysis (see Methods section). As a result, we found that the visual directions (i.e., visual representation) were decoded from the occipital area (p_FWE_ < 0.05, corrected for the whole brain, *n* = 22, one-sided, TFCE), corresponding to the well-known role as a visual area. See Figure 6 for the results of the visual direction decoding analysis. In contrast, the movement directions were decoded from various brain regions (p_FWE_ < 0.05, corrected for the whole brain, *n* = 22, one-sided, TFCE), such as M1, BG, PM, cerebellum, SMG (supramarginal gyrus), DLPFC, ACC, temporal area, and thalamus. These findings imply that a complex interplay of diverse cognitive processes, spanning from implicit to explicit, contributes to generating proper movements during multi-context visuomotor-adaptation tasks. See Figure 7 for the results of the movement direction decoding analysis.

**Figure 8:**
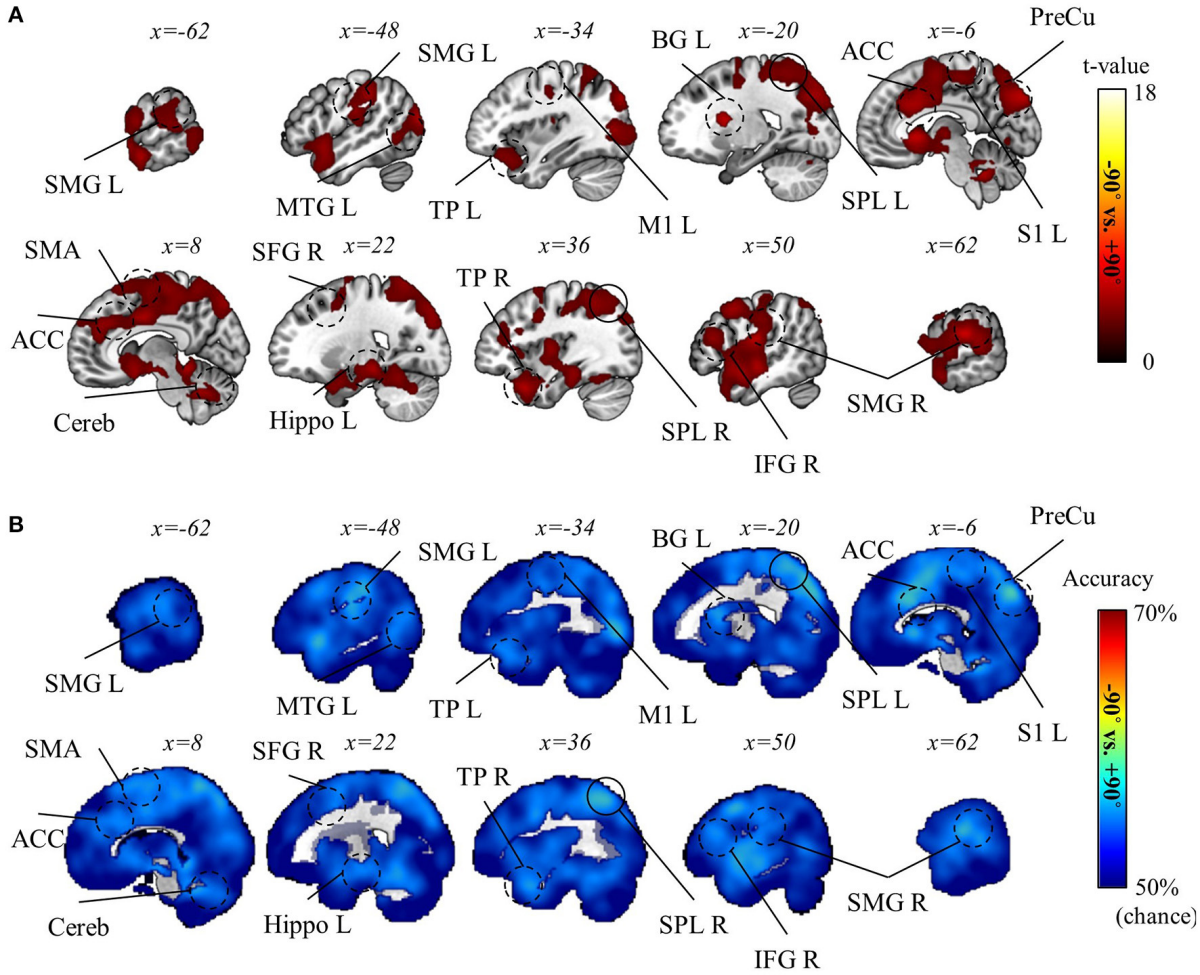
Task-context (−90° vs. +90° rotations) decoding results. **(A)** A t-value map from the group-level analysis of searchlight decoding analysis for task-context representations, −90° vs. +90° rotations, is presented. Only significant voxel t-values are displayed (p_FWE_ < 0.05, corrected for the whole brain, *n* = 22, one-sided, TFCE). **(B)** A subject-averaged accuracy map from the group-level analysis of searchlight decoding analysis for task-context representations, −90° vs. +90° rotations, is presented. Only accuracies that are greater than chance are displayed (>50%). PreCu, precuneus; SPL, superior parietal lobule; SMG, supramarginal gyrus; M1, primary motor area; SMA, supplementary motor area; S1, primary sensory area; Cereb, cerebellum; BG, basal ganglia; IFG, inferior frontal gyrus; ACC, anterior cingulate cortex; Hippo, hippocampus; MTG, middle temporal gyrus; TP, temporal pole; L, left; R, right.

The decoding analysis for task-context representations was done with caution, given the considerable difference in behavioral performance between rotation and mirror contexts (see Behavior results). Therefore, we performed two separate analyses: one decoding the two rotation contexts (−90° and +90°) and the other decoding rotation and mirror contexts (i.e., −90° vs. mirror and +90° vs. mirror). In the latter, the absolute value of performance difference between rotation and mirror contexts was included as a confounding variable to regress out a factor attributable to the performance disparity between rotation and mirror (See Methods section for details). As a result, we discovered that the two rotation contexts were significantly distinguishable in various regions of the brain, such as precuneus, superior parietal lobule (SPL), SMG, BG, M1, SMA, ACC, middle temporal gyrus (MTG), temporal pole (TP), hippocampus, and cerebellum (p_FWE_ < 0.05, corrected for the whole brain, *n* = 22, one-sided, TFCE; Figure 8). However, we could not find any significant result for the decoding analysis for rotation vs. mirror. Despite no significant result, this does not necessarily mean that the brain does not encode task-context representations differentiating these two contexts. The lack of a significant finding may be due to the strict control of the confounding factor (i.e., performance difference). Therefore, we proceeded to perform a more careful examination (i.e., ROI analysis) of this decoding analysis for rotation vs. mirror.

**Figure 9:**
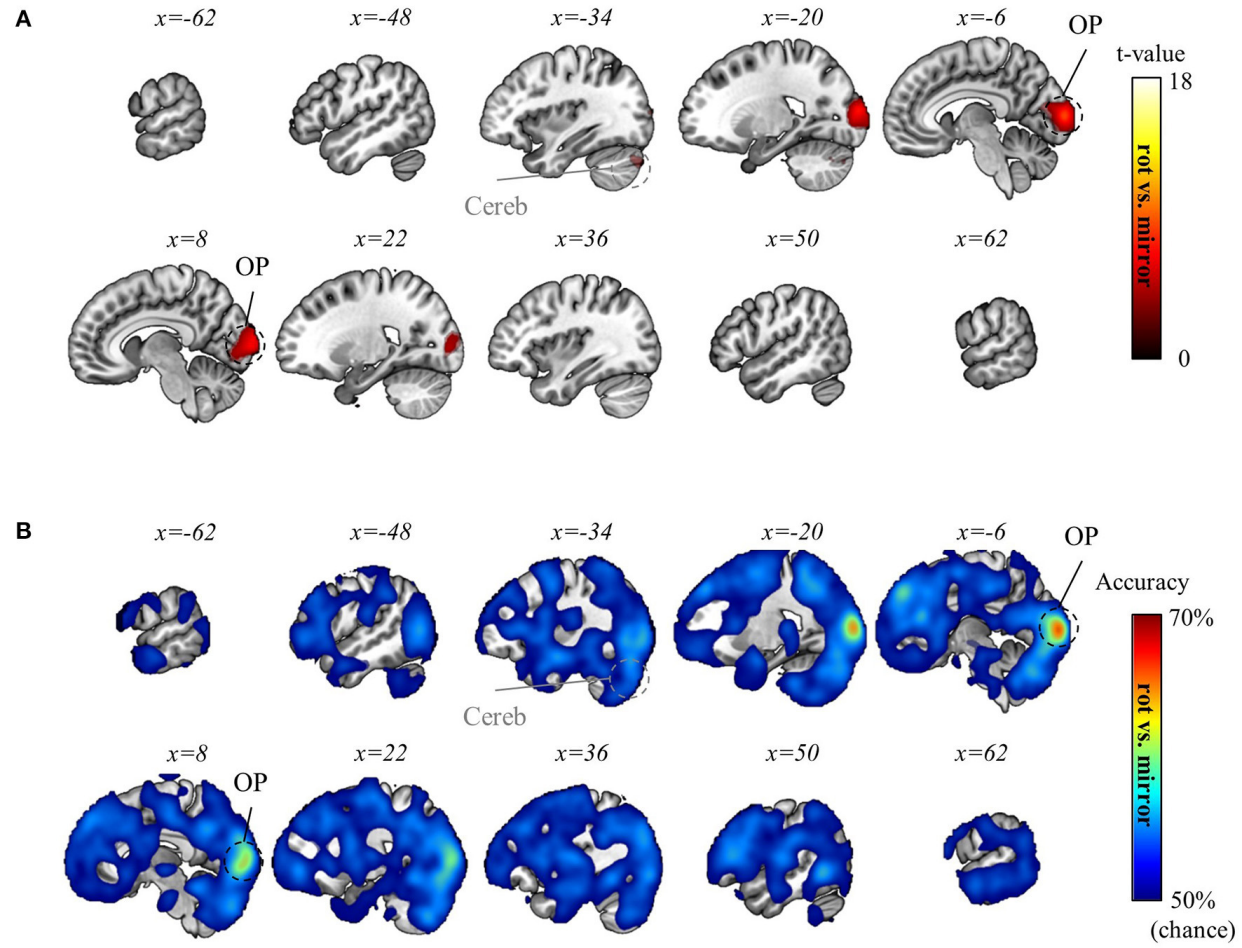
Task-context (rotation vs. mirror) decoding using the preparation-period data. **(A)** A t-value map from the group-level analysis of searchlight decoding analysis for task-context representations (in particular, rotation vs. mirror) using the preparation-period data is presented. T-values of significant voxels from two distinct multiple comparison controls are displayed: one is weakly controlled (i.e., FDR < 0.05) and the other is strictly controlled (i.e., p_FWE_ < 0.05). Both are corrected for the whole brain (n = 22, one-sided, TFCE). The weakly controlled voxels (FDR < 0.05) are depicted as transparent with gray-colored region labels, while the strictly controlled voxels (pFWE < 0.05) are depicted as opaque with black-colored region labels. **(B)** A subject-averaged accuracy map from the group-level analysis of searchlight decoding analysis for task-context representations (in particular, rotation vs. mirror) using the preparation-period data is presented. Only accuracies that are greater than chance are displayed (>50%). No significant voxel was found for “−90° vs. +90° rotations” decoding using the preparation-period data. OP, occipital pole; Cereb, cerebellum.

To this end, we next examined the possibility of task-context differentiation during the preparation period to investigate the brain regions where representational differences emerge in preparation for appropriate visuomotor mapping (Note that the previous context-decoding analysis was done using the BOLD signal during erasing.). To this end, we conducted a searchlight decoding analysis utilizing the signal from the preparation period (see Methods section). As a result, we discovered that rotation and mirror contexts were distinguishable in OP (p_FWE_ < 0.05, corrected for the whole brain, *n* = 22, one-sided, TFCE), whereas no significant result was found for the two rotation contexts (−90° and +90°). The results are illustrated in Figure 9. For a more in-depth understanding of the topological distribution of regions differentiating the contexts, Figure 9 displays voxels with weak control for multiple comparisons (i.e., FDR < 0.05, corrected for the whole brain, *n* = 22, one-sided, TFCE). This suggests that the cerebellum might also differentiate between the rotation and mirror contexts during the preparation period.

Then, we hypothesized that the same regions (i.e., OP and cerebellum) differentiate between the two contexts during the execution of the task. Thus, we conducted an ROI analysis for the OP and cerebellum using the erasing trial data. Within the ROIs that shows significant result in the decoding analysis utilizing the data from the preparation period, a t-test was performed with the absolute value of performance differences between rotation and mirror contexts as a confounding variable (Specifically, we employed a general linear model analysis with two regressors: a constant regressor and the absolute value of the performance difference between the rotation and mirror contexts. Then, our statistical analysis was focused on the constant regressor.). As a result, we found significant results in the OP (t = 2.7390, *p* = 0.0063, one-sided, ROI: the area in the OP, displaying significant decoding results from the preparation data, as shown in Figure 9) and the cerebellum (t = 3.9781, *p* = 0.0004, one-sided, ROI: the area in the cerebellum, displaying significant decoding results from the preparation data, as shown in Figure 9). This suggests that the OP and cerebellum also encode task-context representations regarding rotation and mirror structures while performing the task (i.e., erasing).

### Task-context representation commonality correlates to task performance

Finally, we sought to investigate the impact of task-context representation commonality on task performance. To achieve this, we conducted a correlation analysis between behavioral task performance and task-context level decoding accuracy, with the understanding that high representation commonality may result in low decoding accuracy. This correlation analysis was also split into two parts as the previous context-decoding analysis: one examining the correlation between the mean performance of rotation contexts and the decoding accuracy of the two rotation contexts (−90° and +90°) and the other examining the correlation between mirror context performance and decoding accuracy of rotation and mirror contexts (i.e., −90° vs. mirror and +90° vs. mirror), including the absolute value of performance difference as a confounding variable (see Methods section).

**Figure 10:**
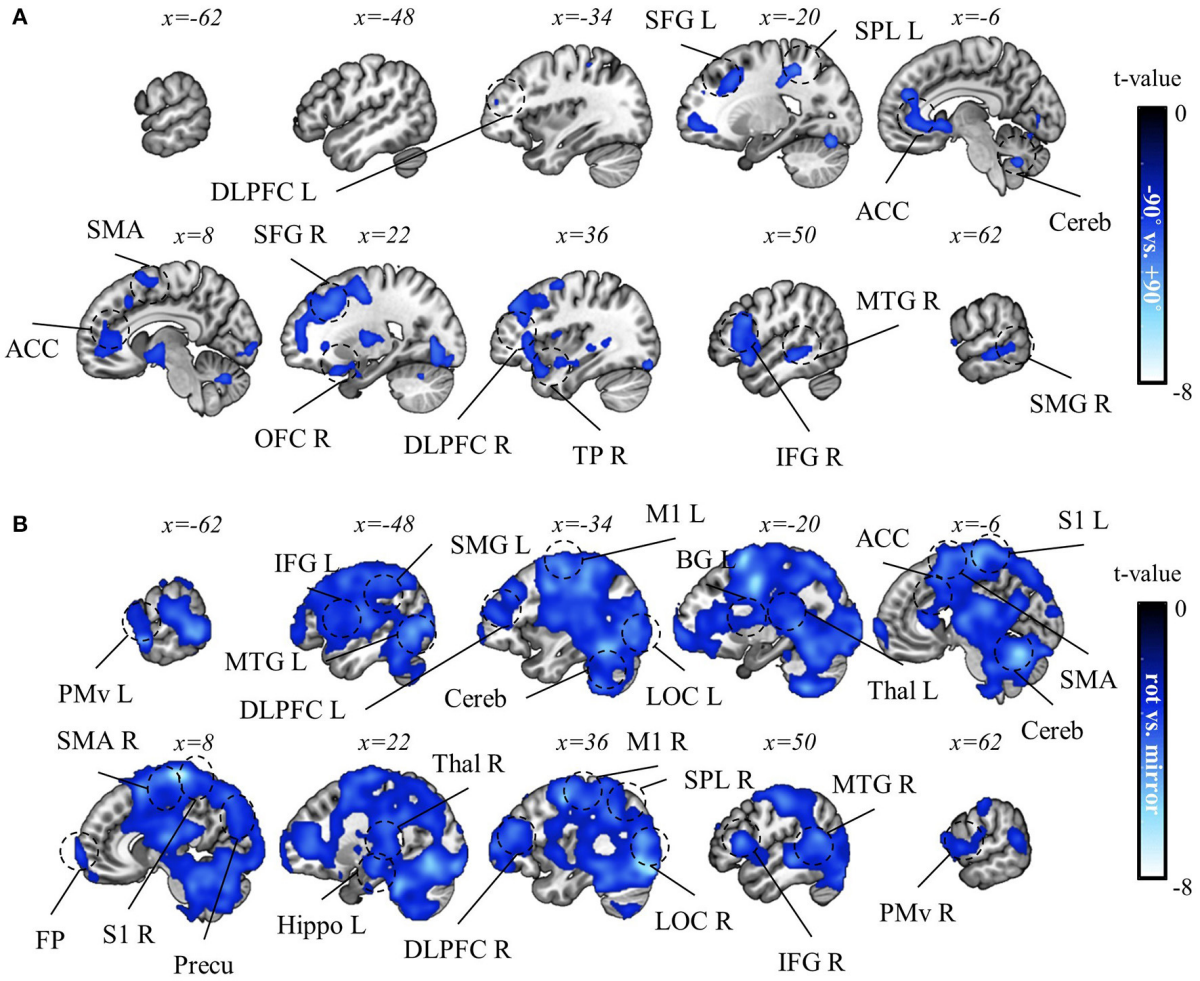
Correlation analysis results between task performance and decoding accuracy for task context. **(A)** A t-value map from the correlation analysis between decoding accuracy for “−90° vs. +90° rotations” and mean rotation-context performance. **(B)** A t-value map from the correlation results between decoding accuracy for “rotation vs. mirror” and mean mirror context performance. For both maps, only significant voxel t-values are displayed (p_FWE_ < 0.05, corrected for the whole brain, *n* = 22, one-sided, TFCE). Notably, no significant positive correlations were observed in this analysis. LOC, lateral occipital cortex, PreCu, precuneus; SPL, superior parietal lobule; SMG, supramarginal gyrus; M1, primary motor area; PMv, ventral premotor area; SMA, supplementary motor area; S1, primary sensory area; Cereb, cerebellum; BG, basal ganglia; Thal, thalamus; DLPFC, dorsolateral prefrontal cortex; IFG, inferior frontal gyrus; ACC, anterior cingulate cortex; OFC, orbitofrontal cortex; FP, frontal pole; MTG, middle temporal gyrus; TP, temporal pole; L, left; R, right.

Consequently, we identified a negative correlation between mean rotation context performance and decoding accuracy of the two rotation contexts in various brain regions, such as PM, M1, SMA, SFG (superior frontal gyrus), ACC, DLPFC, MTG, TP, and cerebellum (p_FWE_ < 0.05, corrected for the whole brain, *n* = 22, one-sided, TFCE; Figure 10A). Also, a negative correlation between mirror context performance and decoding accuracy for rotation vs. mirror contexts was observed in various brain regions, such as OP, LOC, S1, M1, PM, SMA, PPA, BG, thalamus, hippocampus, temporal area, frontal pole, and DLPFC (p_FWE_ < 0.05, corrected for the whole brain, *n* = 22, one-sided, TFCE; Figure 10B). These negative correlations correspond to what we hypothesized based on structural learning: The high context representation commonality (indicated by low decoding accuracy) is associated with higher task performance.

## Discussion

In the current study, we set out to unravel the complexities of visuomotor adaptation, particularly focusing on the neural representations that govern these processes across multiple contexts. Our conjunctional analysis demonstrates that various brain regions participate in processing visuomotor information while encoding distinct levels of representation (i.e., visual, movement, and task contexts) necessary for multi-context visuomotor adaptation. This successful distinction leads to the main finding in this study, which is that the commonality of task contexts (signified by low decoding accuracy) positively correlates with multi-task performance, suggesting the potential advantages of structural learning. These findings provide significant insights into how the brain processes contextual information to perform multi-context visuomotor adaptation tasks efficiently. We will discuss these insights for this efficient visuomotor process more thoroughly, focusing on the following key aspects: (1) structural learning in visuomotor adaptation, (2) structural learning between task contexts with limited shared structure and implications of specific brain regions on structural learning in visuomotor adaptation, (3) differentiating rotation from mirror structure and distinguishing rotations angles, (4) the indication of interplay between context representation and other levels of representation, and (5) limitations of the study.

### Structural learning in visuomotor adaptation

Structural learning constitutes a process through which a system acquires general representations governing a set of contexts. This means that the representations remain common and invariant across contexts, thereby effectively minimizing the dimensionality of the exploratory parameter space required for adapting to mutable environments via the use of common information (Kemp and Tenenbaum, 2008; Braun et al., 2009, 2010a). An instance of this can be understood in the two visuomotor rotation contexts (i.e., −90° and +90°) undertaken in this study. The pivotal common structure, in this case, is rotation. Given that structural learning occurred, the learning of these two task contexts could lead to the extraction of a shared representation, potentially indicative of the rotation structure. As such, the brain, when transitioning between the two rotation contexts, does not necessitate an adjustment of entire internal parameters to establish appropriate visuomotor mappings. Rather, the adjustment of a single parameter—the rotation angle—is sufficient since the common representation (in this instance, the rotation structure) is utilized. This common information reduces the dimensionality of the problem and amplifies learning efficiency. Consequently, it can expedite the learning process, reduce interference, enhance the transferability of tasks, and essentially facilitate a “learning to learn” mechanism (see Braun et al., 2010a, for a thorough review).

Previous studies (Braun et al., 2009; Genewein et al., 2015) indeed showed that structural learning regarding the rotation structure can occur, resulting in such positive effects in multi-context visuomotor learning. The current analysis provides the neural evidence for this phenomenon. In this study, a negative correlation was observed between rotation context performance and context-decoding accuracy (−90° vs. +90°) in various brain areas (Figure 10). This finding can be interpreted as representational commonality associated with multi-task performance since a common representation between two task contexts can result in low context-decoding accuracy due to their insignificant difference. This interpretation supports that structural learning takes place during visuomotor adaptation. This means that if structural learning took place during visuomotor adaptation, a common representation is extracted, positively influencing multi-task performance. To fully understand this observation, it is crucial to consider and address potential alternative explanations to confirm whether the negative correlation truly stems from the positive effects of structural learning.

One potential alternative explanation for the observed results (i.e., the negative correlations between task performance and context-decoding accuracy) is that distinct information processes—requiring distinct representations for the four erasing directions (i.e., up, down, left, and right) within each task context—minimize interference, thereby enhancing performance (Musslick et al., 2017; Musslick and Cohen, 2019; Sagiv et al., 2020; Badre et al., 2021). When partitioning the training and test sets in the context-decoding analysis, data from the same context were partitioned in a manner that ensured the same visual or movement directions were not present in both training and test sets, preventing the decoding from depending on visual or movement directional information (see Methods section). Thus, differing direction-specific representations within the same context could lower decoding accuracy as visual or movement directions vary between the training and test sets. For instance, training to decode task contexts with upward erasing data and testing with rightward erasing data could result in low accuracy if upward erasing in the +90° rotation differs from rightward erasing in the +90° rotation. Then, lower decoding accuracy might be associated with better task performance if these distinct processes and representations for each erasing direction are advantageous for behavior. Under this possibility, decoding accuracy of the four directions within each context should positively correlate with task performance. However, we found no significant positive correlation in ROI analysis at the same brain areas (correlation between decoding accuracy of the four directions within each ROI in −90° context and mean −90° context performance: *p* > 0.2 for all ROIs; correlation between decoding accuracy of the four directions within each ROI in +90° context and mean +90° context performance: *p* > 0.1 for all ROIs; *n* = 22, ROI mask: the clusters that showed the significant negative correlation between rotation context performance and “−90° vs. +90°” decoding accuracy as shown in Figure 10A). Therefore, we speculate that this explanation is unlikely to account for the observed findings.

Another possible explanation for the results is that those areas of well-performed participants may be less involved in the sensorimotor process because the brain economizes resources as learning progresses (Dayan and Cohen, 2011). For instance, the explicit learning implemented by the prefrontal area can be reduced as the participant becomes more proficient, resulting in low BOLD activation as the task proceeds (Floyer-Lea and Matthews, 2004, 2005). Under this possibility, the BOLD signal in these regions should be negatively correlated with task performance since the region of a wellperformed participant shows reduced neural activity. However, we found no significant negative correlation in ROI analysis at the frontal area (correlation between mean BOLD signal within each ROI in −90° context and mean rotation performance: *p* > 0.2 for all ROIs; correlation between mean BOLD signal within each ROI in +90° context and mean rotation performance: *p* > 0.5 for all ROIs; *n* = 22, ROI mask: the clusters that showed the significant negative correlation between rotation context performance and “−90° vs. +90°” decoding accuracy as shown in Figure 10A). Therefore, we speculate that this explanation is also unlikely to account for the observed findings.

Taken together, the negative correlation found in this study is likely attributed to the beneficial impact of common context representations on task performance, where the common representations are potentially derived from structural learning. Following this interpretation, we speculate that each area that shows a negative correlation (as shown in Figure 10A) could play a specific role in extracting the common structure between the rotation contexts (i.e., rotation). Nonetheless, it is noteworthy that the rotation contexts have shared attributes beyond just rotation. For instance, the sensitivity of the computer mouse or the viewing angle of the computer screen can be shared between the rotation contexts due to the consistent use of the same computer mouse and screen throughout. However, these are attributes also present in the mirror context, prompting further discussion in the “rotation vs. mirror” decoding analysis to understand the role of each area when extracting the common task structure.

### Structural learning between task contexts with limited shared structure, and implications of brain regions regarding structural learning in visuomotor adaptation

Given that the structure of the mirror-reversal context shares only limited similarities with visuomotor rotation contexts, a common representation might hinder performance by promoting interference instead of mitigating it (Musslick et al., 2017; Musslick and Cohen, 2019; Sagiv et al., 2020; Badre et al., 2021). However, the negative correlation between mirror context performance and context-decoding accuracy (rotation vs. mirror) was still observed in the various brain regions ranging from the posterior to the anterior regions (Figure 10B). We confirmed that the alternative explanations previously discussed are also unlikely to explain this negative correlation (i.e., we could not find any significant voxel in the correlation analysis between decoding accuracy of the four directions in mirror context and mean mirror context performance and the correlation analysis between the mean BOLD signal in mirror context and mean mirror context performance: corrected within the ROI, cluster-level correction with uncorrected threshold *p* < 0.001, *n* = 22, one-sided, random-field theory, ROI mask: all the areas that showed the significant negative correlation between mirror context performance and “rotation vs. mirror” decoding accuracy as shown in Figure 10B). This finding is an indication that structural learning can occur and benefit performance by extracting the shared task structure even between rotation and mirror contexts.

As discussed above, computer mouse sensitivity and the screen's viewing angle can be shared between rotation and mirror contexts due to the consistent use of the same computer mouse and screen. Consequently, fine adjustments to mouse sensitivity and the processing of sensory data (whether visual or somatosensory) for task-specific details (like the mouse cursor's position) are shared elements of both task contexts. The former process can be supported by the cerebellum (Martin et al., 1996; Krakauer et al., 2019; Markov et al., 2021; Tzvi et al., 2022); while the latter can be supported by the LOC and PPA (Husain and Nachev, 2007; Sack, 2009). Therefore, the negative correlation in the cerebellum, LOC, and PPA is a possible indication that creating common representations for those processes can enhance task performance. Moreover, due to the environmental consistency between rotation and mirror contexts, it can be beneficial to have common representations for modulating voluntary muscle movements by M1, coordinate planning by PM or SMA, or regulating movement amplitude and velocity by BG (DeLong et al., 1984a,b; Pearson, 2000; Graziano, 2006; Dudman and Krakauer, 2016; Krakauer et al., 2019; Bear et al., 2020; Bhattacharjee et al., 2021). Thus, negative correlations in such motor areas may implicate those benefits.

Interestingly, the anterior region, such as the frontal pole, showed a negative correlation between mirror context performance and context-decoding accuracy, suggesting that the more abstract and explicit aspects of the cognitive process can be facilitated by building a common representation, ultimately leading to being more proficient at performing both task contexts (Koechlin and Summerfield, 2007; O'Reilly, 2010). Given the observed correlation in the thalamus, hippocampus, and frontal pole and the implications of such areas in task-context inference (Heald et al., 2023), the negative correlations in those areas may indicate that optimizing those processes by building common representation can benefit the process of such multi-contextual inference. Future research needs to be addressed to gain a deeper understanding of the role of those areas.

To pinpoint the regions responsible for extracting rotation structure, we then focused on areas that demonstrated a significant correlation in −90° vs. +90° decoding (p_FWE_ < 0.05) as shown in Figure 10A and lacked significance in the rotation vs. mirror decoding as shown in Figure 10B, even with the weak control of multiple comparisons (FDR > 0.05). Areas such as SFG, IFG, TP, and MTG met these criteria, suggesting their vital roles in extracting rotation structure. We note that the rotation structure shared by the two rotation contexts is a highly abstract aspect of task rules (i.e., mapping between sensory input and proper motor output), requiring the encoding and extraction of these abstract components to facilitate structural learning. Thus, the commonality of neural representation in the frontal areas (i.e., SFG and IFG) suggests the frontal representation of such an abstract aspect of visuomotor mapping, underscoring the role of the frontal area in abstract rule-based learning (Badre and D'Esposito, 2007; Eiselt and Nieder, 2013). This implication is highly related to the role of the frontal area in fostering cognitive flexibility in sensorimotor control (Keisler and Shadmehr, 2010; Taylor and Ivry, 2014; McDougle et al., 2016). Given the temporal cortex's recognized role in high-order functions such as processing abstract concepts or semantic memories (Binder et al., 2009; Chaumon et al., 2009; Binder and Desai, 2011), the common neural representations in temporal regions, such as TP and MTG, might suggest their role in representing the abstract or semantic dimension of rotation structure. Future research should explore how the frontal and temporal regions contribute to extracting such abstract task information and flexibly applying it to different contexts.

Overall, our findings imply that structural learning can occur at multiple structural levels, ranging from high-level abstractions (e.g., rotation structure) to lower-level details (e.g., viewing angle), indicating that the brain optimizes the advantages of multi-context learning by extracting as much commonality as possible. These findings highlight the brain's efficient learning mechanisms, which dynamically balance multiple structural levels to optimize performance. As a result, it raises intriguing questions about the extent to which various structural levels impact the overall effectiveness of multi-context learning, which should be addressed in future studies.

Although we did not find any positive correlation between mirror context performance and context-decoding accuracy, our study does not rule out the possibility that a common context representation might still cause confusion and hinder performance. To truly understand this, we may need to conduct experiments with tasks that are very different from one another. This would allow the interference to become the dominant factor, making it easier to observe than structural learning, which should also be explored in future research.

### Differentiating rotation from mirror structure and distinguishing rotations angles (−90° vs. +90°)

Context can be characterized as a distinct variable that governs the active set of contingencies among potentially numerous alternatives; specifically, it defines the associations among sensory inputs, environmental states, and their dependence on actions (Heald et al., 2022). This characterization aligns with the task context examined in this study, where the task contexts (i.e., −90° rotation, +90° rotation, and mirror-reversal) dictate the relationship between potential sensory inputs (i.e., visual direction of a given stimulus) and appropriate motor outputs (i.e., hand-movement direction). Thus, although the brain employs structural learning at multiple structural levels and extracts as much commonality as possible to optimize the multi-context information process, the brain still needs to distinguish the three task contexts (i.e., −90° rotation, +90° rotation, and mirror-reversal) to generate proper movements in each task context. Our findings in the context-decoding analysis provide insights into this process.

As observed in Figure 9, the OP and possibly the cerebellum significantly distinguish between rotation and mirror contexts during both the execution and preparation of such tasks (see Results for detail). Drawing from prior research, the OP is known to play a role in visualizing tasks (Albers et al., 2013; Koenig-Robert and Pearson, 2019); meanwhile, the cerebellum is involved in predicting the outcomes or consequences of our actions (Wolpert et al., 1998; Krakauer et al., 2019; Tanaka et al., 2020). Thus, our observation implies that the task visualization by the OP and action–outcome prediction by the cerebellum seem to operate differently depending on the task context. In other words, how the brain visualizes and predicts outcomes for rotation tasks is distinct from how it does so for mirror tasks. This distinction is crucial, as it helps in establishing the correct sensory-motor connections appropriate for each type of context with largely different task structures.

Although many brain areas are implicated in differentiating the two rotation angles of rotation contexts (i.e., −90° vs. +90°) (Figure 8), most of these areas also display negative correlations between the mean performance of rotation contexts and the decoding accuracy of the two rotation contexts (Figure 10A). This suggests that in most areas, the common representation of the rotation structure is yet to be constructed. In other words, these areas might not be essential for differentiating the rotation angles once the common representation is established. However, a notable difference was observed in the PPA (which includes SPL and precuneus) when comparing Figures 8A, 10A: This area significantly distinguishes the two rotation contexts but lacks a negative correlation concerning task performance. This strongly indicates that the PPA is involved in differentiating the rotation angles (i.e., −90° vs. +90°) rather than encoding the rotation structure shared by the two rotation contexts. The role of the PPA in distinguishing the rotation angle in visuomotor adaptation was also noted in a previous MVPA study (Haar et al., 2015). Moreover, the PPA is known for its role in integrating sensory information and forming spatial representations necessary for coordinating movements in space (Husain and Nachev, 2007; Sack, 2009), and importantly, this area is associated with mental rotation (Jordan et al., 2001; Gogos et al., 2010). Thus, our finding highlights the significance of PPA in such spatial reasoning regarding the abstract aspect (i.e., rotation angle) and the possible involvement of mental rotation in the visuomotor rotation task.

### The indication of interplay between context representation and other levels of representation

The sensorimotor process in the brain can be described as a mapping from sensory inputs to motor outputs (i.e., sensorimotor mapping) (Pouget and Snyder, 2000; Franklin and Wolpert, 2011; Merel et al., 2019). Thus, the contextual process can be described as a higher-order process governing this mapping. One last notable observation in this study is that the regions highlighted in Figures 8–10, which display these context-related areas, demonstrate significant overlap with either the visual representation areas depicted in Figure 6 or the movement representation areas depicted in Figure 7. This overlap suggests a potentially integral relationship between visual and movement representations with context-dependent brain areas. In other words, such a pattern suggests that the proper encoding of movement direction is not just a straightforward sensorimotor transformation but may also be influenced by the specific task contexts in which movements are made.

The visual representation (i.e., the direction of a given line on the screen) can be decoded in the occipital areas, including OP and LOC. This finding aligns with previous research (Haar et al., 2015) and is consistent with the known directional or shape-related selectivity in these areas (Hubel and Wiesel, 1959; Boynton and Hegdë, 2004; Kamitani and Tong, 2005; Larsson and Heeger, 2006; Alink et al., 2013). Notably, the OP not only decodes visual representation but also distinctly differentiates between rotation and mirror contexts. This suggests that the encoding of visual direction might be influenced by the task context. Given the recognized role of OP in task visualization (Albers et al., 2013; Koenig-Robert and Pearson, 2019), it is plausible that the encoding of visual direction is contingent upon the visualization required by each specific task.

Second, the movement representation (i.e., the direction of hand movement) can be decoded across a range of brain areas, and these areas demonstrate a notable consistency with BOLD-activated brain areas during task performance (excluding visual areas): e.g., M1, PM, SMA, PPA, BG, and cerebellum. These areas align with movement-selective regions identified in previous research (i.e., M1, PM, SMA, and cerebellum; Haar et al., 2015), thereby underscoring the validity of these regions' role in representing movement directions.

Each of these areas delineates a specific aspect of the process of generating appropriate movements. For instance, M1 modulates voluntary muscle movements by producing neural signals, while the PM and SMA coordinate planning and action-sequence preparation to facilitate motor actions (Pearson, 2000; Graziano, 2006; Bear et al., 2020; Bhattacharjee et al., 2021); the PPA contributes to spatial reasoning (Husain and Nachev, 2007; Sack, 2009); the BG, including caudate and putamen, play a vital role in modulating motor functions, such as the initiation and execution of movements and the regulation of movement amplitude and velocity, as well as the cognitive aspect of motor learning (DeLong et al., 1984a,b; Dudman and Krakauer, 2016; Krakauer et al., 2019); the cerebellum's role is to refine movements, analyzing sensory feedback to adjust motor outputs for precision (Martin et al., 1996; Krakauer et al., 2019; Markov et al., 2021; Tzvi et al., 2022); and lastly, the DLPFC is implicated in selecting and maintaining task-relevant information and monitoring and adjusting motor performance based on feedback and task demands, as well as strategic modifications in aiming (Anguera et al., 2007, 2009; Seidler et al., 2012; McDougle et al., 2016).

In summary, the movement directional selectivity distributed across various brain regions suggests a multi-faceted approach to executing appropriate movements. These processes range from cognitive aspects, such as explicit aiming, to more autonomous mechanisms, such as the fine-tuning of movements. The notable overlap of these movement-selective areas with context-related areas suggests that the multi-faceted process (ranging from cognitive to autonomous aspects) may be largely dependent on specific task contexts, shedding light on the intricate relationship between sensory input interpretation and its translation to motor action under diverse task conditions.

Moreover, M1, PM, SMA, and PPA are also activated during the preparation period, signifying their role in preparing sensorimotor mapping and comprehending upcoming context (Brass and Von Cramon, 2002; Fontana et al., 2012; Bhattacharjee et al., 2021). These observations underscore the significance of these areas' functional roles in multi-context processes. Conversely, BG, cerebellum, and DLPFC were activated exclusively during performing tasks and not during the preparation phase. These findings suggest their distinct involvement in real-time motor control and learning, which are potentially associated with performance monitoring and adjustment. Yet, understanding the exact contributions of task-context representations in these regions to the visuomotor process remains an open question for future studies.

### Limitations of the study

Although the current study has provided valuable insights, it is crucial to recognize its limitations and identify potential directions for future research. First, the tasks utilized in this study may not fully capture the complexity of real-world visuomotor adaptation or the neural representation of visual directions: for example, being limited to four erasing directions or necessitating brief erasing movements (i.e., erasing a short line). While the current experimental paradigm offers controlled conditions for data collection, it may not adequately represent the complexities of everyday visuomotor tasks that individuals typically face. Developing more ecologically valid tasks that emulate real-life situations could enhance our understanding of the underlying neural mechanisms and increase the generalizability of the findings. Future studies could explore different task designs that account for the dynamic nature of real-world visuomotor adaptation, incorporating a wider variety of task conditions and difficulty levels.

Second, we limited our analysis to using a linear classifier (i.e., SVM) for multivariate decoding analysis; as a result, the current study might have identified only some neural representations during visuomotor adaptation. Although linear separability is a crucial foundation for understanding neural representation, non-linear classifiers, such as deep learning or kernel methods, can offer a more comprehensive understanding of the sensorimotor process. Consequently, future research should consider employing more comprehensive techniques, including non-linear classifiers, to validate the present findings and investigate the robustness of the observed neural representations of visual directions during visuomotor adaptation tasks.

## Conclusion

In conclusion, our study emphasizes the brain's efficient learning mechanisms by extracting commonality at various task-structural aspects, and the intricate interplay between context representations and the encoding visual and movement representations. This study raises intriguing questions about the extent to which various structural levels impact the overall effectiveness of multi-context learning and sets the stage for future investigations into the precise mechanisms through which the brain extracts and applies abstract task information to different contexts. Future research should continue to investigate the brain's intricate but efficient nature and the implications of these findings for real-world applications.

## Data availability statement

The datasets generated for this study can be found in the OpenNeuro under the accession number ds004562. The code used to perform the experiments and analyze the data is available at GitHub (https://github.com/YJ-0000/Multi-context_Representation_during_VisuomotorAdaptation.git).

## Ethics statement

The studies involving humans were approved by Institutional Review Board of the Korea Advanced Institute of Science and Technology. The studies were conducted in accordance with the local legislation and institutional requirements. The participants provided their written informed consent to participate in this study.

## Author contributions

YS conceptualized the study, performed the experiments, analyzed the experimental data, interpreted the results, and wrote the manuscript. WS performed the experiments and revised the manuscript. PK revised the manuscript. JJ acquired funding, administrated the project, and supervised the study. All authors contributed to the article and approved the submitted version.
